# Multi-Site Tumour Sampling Improves the Detection of Intra-Tumour Heterogeneity in Oral and Oropharyngeal Squamous Cell Carcinoma

**DOI:** 10.3389/fmed.2021.670305

**Published:** 2021-05-10

**Authors:** Weiping Jie, Jiaying Bai, Jing Yan, Yanting Chi, Bin-bin Li

**Affiliations:** ^1^Department of Oral Pathology, Peking University School and Hospital of Stomatology, National Clinical Research Center for Oral Diseases, National Engineering Laboratory for Digital and Material Technology of Stomatology, Beijing Key Laboratory of Digital Stomatology, Beijing, China; ^2^Research Unit of Precision Pathologic Diagnosis in Tumours of the Oral and Maxillofacial Regions, Chinese Academy of Medical Sciences, Beijing, China

**Keywords:** multi-site tumour sampling, intra-tumour heterogeneity, oral squamos cell carcinoma, histology, immunohistochemistry, gene mutation, gene methylation, oropharyngeal squamous cell carcinoma

## Abstract

**Background:** Oral squamous cell carcinoma (OSCC) and oropharyngeal squamous cell carcinoma (OPSCC) are very common in head and neck malignancy. Intratumour heterogeneity (ITH) may hamper their responses to treatment. Hence, novel tumour sampling methods that reflect ITH are required. In this study, we investigated the clinical significance of multi-site tumour sampling (MSTS) to detect ITH in OSCC and OPSCC.

**Methods:** One hundred eighty-two paired specimens were sampled by routine sampling (RS) or MSTS, respectively. Histologically, tumour grade, peri-tumoural vascular and lymphatic growth, perineural permeation, tumour necrosis, and muscle invasion were assessed. Immunohistochemically, the positive and average detection rates of P53(mutant), ki67 and CyclinD1 were detected. The exon 9 and exon 20 mutations of PIK3CA gene and the methylation status of the CDKN2A promoter were analysed.

**Results:** Microscopically, the detection rate of perineural permeation, the detection density of peri-tumoural vascular and lymphatic growth, necrosis and muscle invasion in MSTS were significantly more frequent than those in RP (*P* < 0.05, *P* < 0.05, *P* < 0.01, *P* < 0.01). MSTS resulted in a higher detection rate of P53 (mutant), ki67, and CyclinD1 expression than did RS, but the difference was not significant. MSTS's detection rates in PIK3CA gene mutation and gene methylation sequencing in CDKN2A gene promoter region were both higher than RP (*P* < 0.05, *P* < 0.01). To be emphasised, the hotspot mutation H1047Rwas detected in one MSTS specimen (case 24M5) but in no RS specimens.

**Conclusions:** This study verified that MSTS's advantage in the reflection of morphological and molecular characteristics of OSCC and OPSCC. MSTS was more representative than RP. Therefore, MSTS can compensate the RP limitations in ITH detection especially in large tumours.

## Introduction

Squamous cell carcinoma (SCC) is a common histological type in oral cavity and oropharynx (1). The mortality rate of these cancers remains poor, owing to its high potential for invasion, maxillofacial destruction, cervical lymph node metastasis, and blood-borne dissemination to distant sites (2). Current treatments include surgery, radiotherapy, chemotherapy, photodynamic therapy, EGFR inhibitors (1), and immunotherapy (3). Although targeted therapies have exerted potent and long-lasting anticancer effects, intratumour heterogeneity (ITH) could lead to treatment failure and therapeutic resistance (4–6). Mounting evidence have suggested that ITH is extremely complex, hampering the wide success of targeted therapies. Spatial heterogeneity is a form of ITH (7, 8). It is common that well-differentiated, poorly differentiated, and moderately differentiated areas can co-exist within an carcinoma. These intra-tumoural differences in differentiation usually lead to varying patterns in lesions, which may cause differential expression of therapeutic targets and influence the treatment response (4).

Genetic heterogeneity always hides behind microscopic homogeneity (4). The PI3K pathway is a well-characterised oncogenic axis (9, 10) that has been associated with drug resistance and poor prognosis (11). In head and neck region, PIK3CA is the third most commonly mutated gene (12), and the PI3K signalling is believed to be a key driver of resistance to EGFR inhibitors (13). Tumours with identical histological features may harbour different PI3K mutations. And the heterogeneity in PIK3CA mutations may play a critical role in the response to targeted therapies.

In addition, epigenetic heterogeneity can be often seen in the genetically identical lesions (4). Notably, promoter methylation can cause gene silencing in the absence of DNA mutations (14), leading to differential expression patterns in tumours with the same DNA sequence. For instance, the promoter of CDKN2A is frequently methylated in HNSCC and other oral cancers (15). Differences in the methylation patterns of CDKN2A promoter may affect the treatment response. An accurate pathological diagnosis of OSCC and OPCC is crucial for clinical decision making and treatment response.

Specimen sampling is the cornerstone of accurate diagnosis and clinical decision making. An limitation of routine sampling (RS) practise is that macroscopically homogeneous tumours may differ in their molecular characteristics due to ITH (16). During RS, only a small portion of the tumour is sampled. Thus, the sample may not represent the entire tumour, especially for large tumours (diameter > 3 cm).

Lopez et al. (16) have developed computer mathematical models to improve tumour sampling methods. They proposed a multi-site tumour sampling (MSTS) method based on the computer “divide and conquer algorithm” and found that MSTS can detect ITH in clear cell renal cell carcinoma (CCRCC) efficiently and economically (17–20). However, the ability of MSTS to detect ITH in OSCC and OPSCC remains unclear (21). In this study, we evaluated the practical usage of MSTS to detect ITH in terms of histology, immunophenotype, mutations, and promoter methylation patterns in OSCC and OPSCC.

## Materials and Methods

### Patients and Specimen Collection

OSCC and OPSCC specimens obtained by radical surgery were collected from the Department of Oral Pathology of the Peking University Hospital of Stomatology (Beijing, China). These tumour masses were from 26 cases, and were made pairs, respectively, based on the different sampling method (described following). The inclusion criteria were as follows: (1) SCC (including the subsite of tongue, gingival, buccal, the floor of the mouth, hard palate, base of tongue and oropharynx) was diagnosed by biopsy, (2) maximum tumour diameter of ≥3 cm; (3) provision of informed consent for potential use of the surgical specimens for scientific research. The study was approved by the Ethics Committee of the Peking University Hospital of Stomatology (No. 2017-09-34-21).

### Tumour Sampling

**Figure 1:**
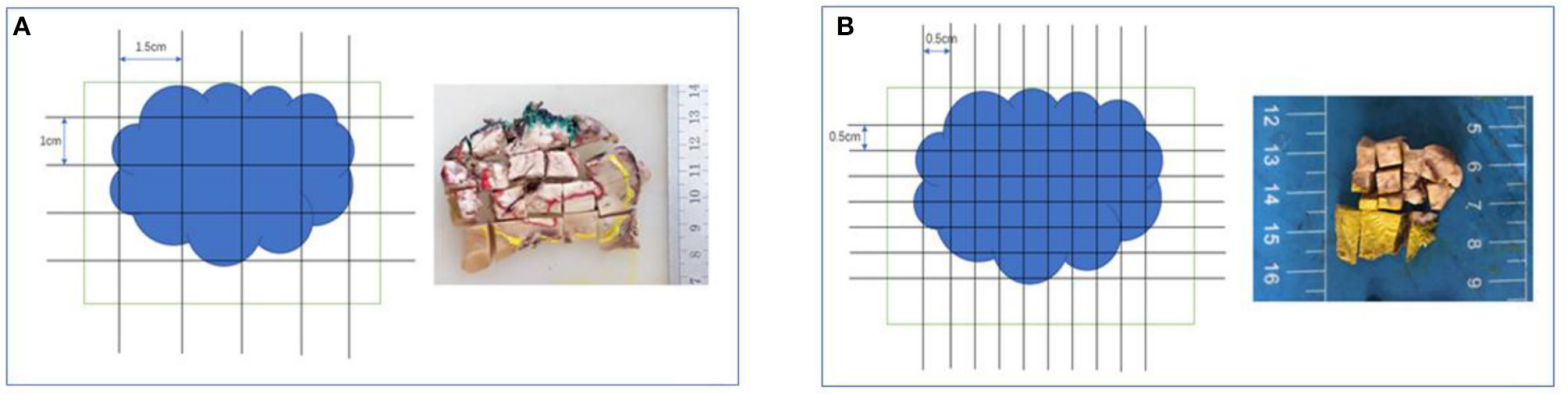
Two methods of tumour sampling. **(A)** Routine sampling, **(B)** multi-site tumour sampling.

**Figure 2:**
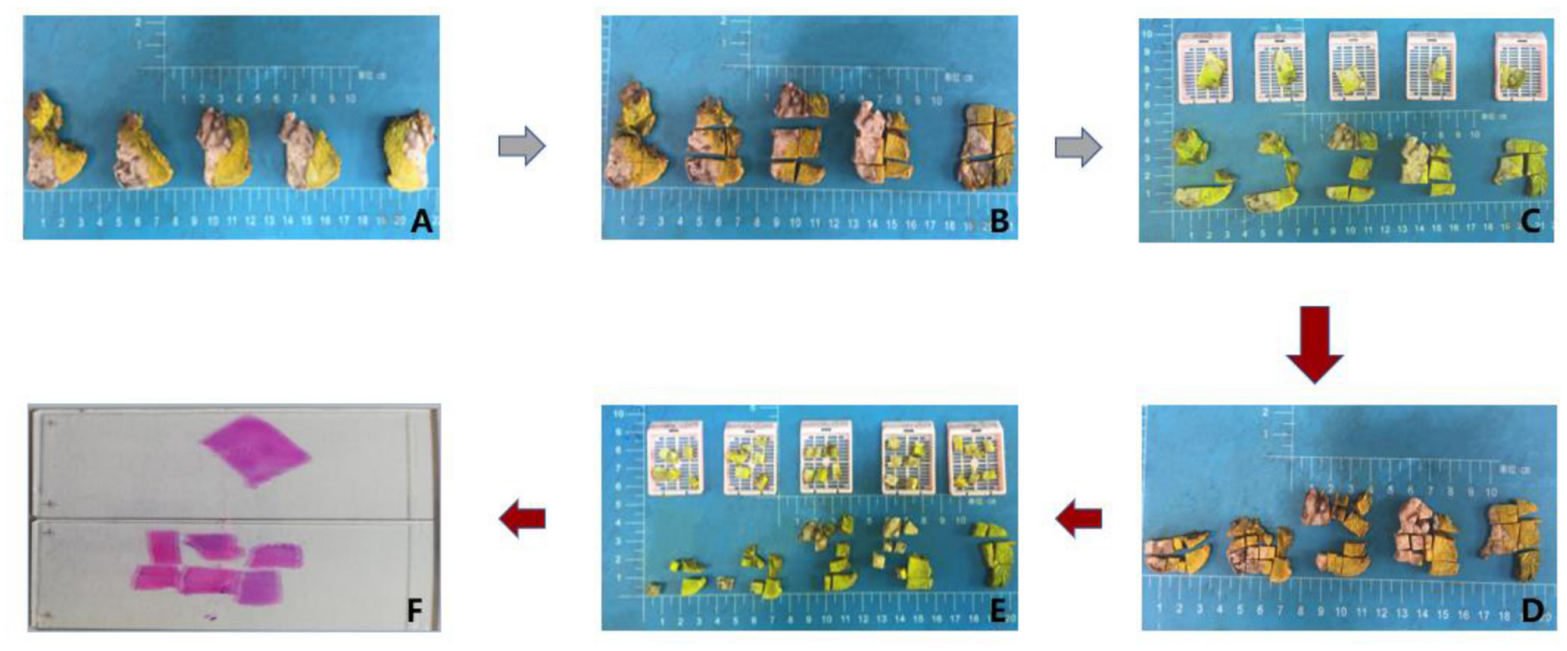
Workflow of tumour sampling. First routine sampling **(A,B,C,F)** and then multi-site tumour sampling **(A–F)**.

To assess the invasion depth, we sampled surgical specimens by excising through the lesion's largest invasion surface with adjacent normal tissues. Specimens were sampled by both RS and MSTS. For RS, after cutting the specimen into parallel cross-sections, we acquired one tissue block as 1.5 cm (length) × 1 cm (width) × 0.3 cm (depth) for each centimetre of mass diameter. For MSTS, we sampled six small scattered tissue blocks [0.5 cm (length) × 0.5 cm (width) × 0.3 cm (depth) each] for each centimetre of mass diameter, and these blocks were used to construct a tissue cassette (Figures 1, 2).

### Hematoxylin and Eosin Staining

Specimens obtained by RS and MSTS were embedded in paraffin and processed routinely. Tissue sections were stained with hematoxylin and eosin and reviewed by an experienced pathologist. Tumour grade, inflammation intensity around the tumour, peri-tumoural vascular and lymphatic growth, perineural permeation, tumour necrosis, and muscle invasion were assessed. The detection density of above parameters was calculated as the total detection number/total detection area of tissues. To simplify the calculation, we considered sampling blocks as mathematical shapes (triangle, square, rectangle, trapezoid) and calculated the area of the sampling block using the corresponding formula.

The inflammation intensity around the tumour was assessed and scored by no inflammation (score 0), mild inflammation (score 1), moderate inflammation (score 2), severe inflammation (score 3).

### Immunohistochemistry (IHC)

Tissue sections were incubated with a ready-to-use primary mouse monoclonal anti-p53 (mutant) antibody (clone DO-7, ZSGB-BIO), mouse monoclonal anti-Ki67 antibody (clone GM-001, Gene Tech), and rabbit monoclonal anti-cyclin D1 antibody (clone EP12, ZSGB-BIO), according to the manufacturers' instructions. Tissue sections were also stained with negative and positive control antibodies. Stained tissues were evaluated by an experienced pathologist. The detection rate of positive IHC expression was calculated as the number of positive cases/total number of cases. The average rate of IHC positivity was calculated as the sum of positive IHC expression percentages in all sample blocks/number of sample blocks.

### DNA Extraction

DNA extraction was performed using the QIAamp DNA FFPE Tissue Kit (QIAGEN, Dusseldorf, Germany) following the manufacturer's instructions. Particularly, DNA was extracted from six small samples in each MSTS tissue cassette, respectively. DNA quality was analysed by 0.8% agarose gel electrophoresis, and the DNA concentration was assessed using the Nanodrop 2000C spectrophotometer (Thermo Fisher, Waltham, USA).

### Mutation Analysis

Exons 9 and 20 of the PIK3CA gene were amplified from genomic DNA by nested polymerase chain reaction (PCR) using a thermocycler (ABI, Carlsbad, USA). The PCR solution contained 100 ng genomic DNA, 10 pmol each primer, 2.5 μL 10 × Taq buffer (with MgCl_2_), and 1 IU Taq Plus DNA Polymerase (Sangon Biotech, Shanghai, China) in a total volume of 25 μL. Samples were preheated at 95°C for 5 min, followed by 38 cycles of 94°C for 30 s, 58°C for 30 s, and 72°C for 60 s; a final elongation step was performed at 72°C for 10 min. The PCR products were sequenced on an autosequencer (ABI, Carlsbad, USA) using the BigDye Terminator Kit (Thermo Fisher, Waltham, USA). Mutations were confirmed by reverse sequencing using the antisense PCR primer and by sequencing a second PCR product from the same sample. The sequences of the primers used for nested PCR and sequencing are shown in Table 1.

**Table 1:** The primers used for nested PCR and sequencing of PIK3CA.

**PIK3CA**	**Forward primer sequence** **(5′- 3′)**	**Reverse primer sequence** **(5′- 3′)**	**Size (bp)**
PIK3CA-9a	ATTATGTCTTAGATTGGTTCTTTCC	GCTTTATTTATTCCAATAGGTATGG	427
PIK3CA-9b	TTGCTTTTTCTGTAAATCATCTGTG	ATAGGTATGGTAAAAACATGCTGAG	304
PIK3CA-20a	GAGCAAAGACCTGAAGGTATTAACA	GTCTTTGCCTGCTGAGAGTTATT	412
PIK3CA-20b	TCCAAACTGACCAAACTGTTCTTAT	GCAGTGTGGAATCCAGAGTGAG	350

### Methylation Analysis

The methylation status of the *CDKN2A* promoter was analysed by bisulfite sequencing PCR. The bisulfite reaction was performed using the EZ DNA Methylation-Gold Kit (ZYMO RESEARCH, Beijing, China) according to the manufacturer's instructions. The bisulfite-modified DNA was amplified by PCR using specific primers. The PCR solution contained 20–50 ng bisulfite-modified DNA, 20 pmol each primer, 30 pmol dNTP, 5 μL 10 × Taq buffer (with MgCl_2_), and 2.5 IU Taq Plus DNA Polymerase (Sangon Biotech, Shanghai, China) in a total volume of 50 μL. Samples were preheated at 95°C for 3–5 min, followed by 35 cycles of 94°C for 30 s, 55–60°C for 25–30 s, and 72°C for 30–50 s; a final elongation step was performed at 72°C for 5–8 min. Reactions were run on the Veriti 96-well-thermocycler. The PCR products were subjected to next-generation sequencing on the MiSeq high-throughput sequencer (Illumina, San Diego, USA). The sequences of the primers used for bisulfite-modified DNA amplification are shown in Table 2.

**Table 2:** The primers used for Bisulfite-modified DNA amplification of CDKN2A.

**CDKN2A**	**Forward primer sequence** **(5′- 3′)**	**Reverse primer sequence** **(5′- 3′)**	**Size (bp)**
	GGTTGGTTATTAGAGGGTGGG	CAATCAACCRAAAACTCCATACTAC	130

### Statistical Analysis

IBM SPSS Statistics 20.0 software was used for all statistical analyses. Differences in histological parameters between the sampling methods were analysed by Student's *t*-test. Differences in the detection rates of different histological parameters, IHC staining results, gene mutations, and methylation status were analysed by Fisher's exact test. Multiple comparisons were performed to determine differences in the average positivity rate of IHC staining among multi-site samples. *P* < 0.05 were considered statistically significant.

## Results

### Patient Characteristics

One hundred eighty-two OSCC and OPSCC specimens with a maximum tumour diameter of ≥3 cm were collected from 26 patients (20 men and 6 women). The median age of the patients was 61 (range, 35–79) years. The most common site of involvement was the mobile tongue and gingiva (23.1%), followed by the floor of the mouth (19.2%), oropharynx (15.4%), and base of the tongue (7.7%). The average maximum tumour diameter was 4.2cm (range, 3–7 cm).

### Histological Characteristics

The test cases were diagnosed as oral squamous cell carcinoma (ICD code: 8070), HPV-negative oropharyngeal squamous cell carcinomas (ICD code: 8086), and HPV-positive oropharyngeal squamous cell carcinomas (ICD code: 8085), respectively. Pathological tumour grades were determined according to WHO classification guidelines for head and neck tumours (2017) (22, 23), including 11 (42.3%) tumours well-differentiated, 10 (38.5%) moderately differentiated, 3 (11.5%) poorly differentiated, and 2 (7.7%) HPV-positive OPSCC. There were no significant differences in the pathological grade and inflammatory response in the samples obtained by RS vs. MSTS. (*P* > 0.05, analysed by Fisher's exact test).

Additionally, all cases (100%) exhibited peri-tumoural vascular and lymphatic growth regardless of the sampling method. However, the rate of vascular density detection was significantly higher in MSTS (4.71 times/cm^2^) than RS (2.44 times/cm^2^) specimens (*P* < 0.05).

**Figure 3:**
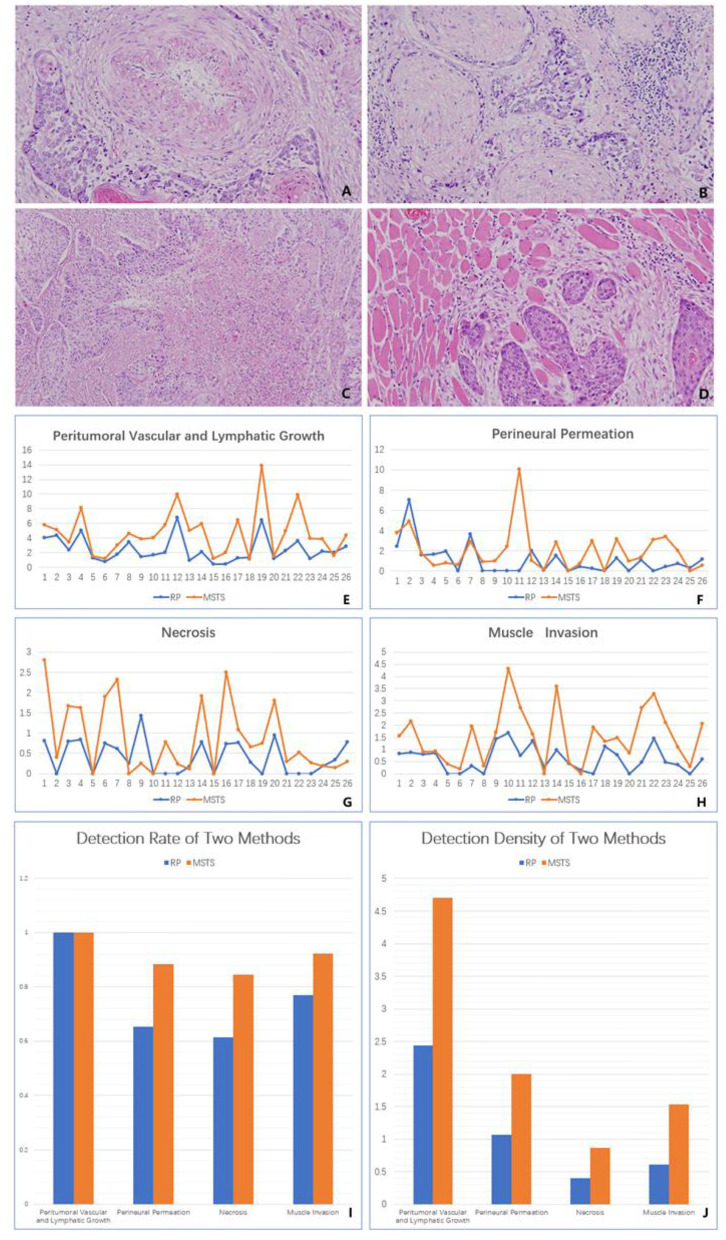
Histology. **(A)** Peritumoral vascular and lymphatic growth (original magnification was 40×). **(B)** Perineural permeation (original magnification was 40×). **(C)** Tumour necrosis (original magnification was 40×). **(D)** Muscle invasion (original magnification was 40×). **(E)** The detection density of peritumoral vascular and lymphatic growth in two methods. **(F)** The detection density of perineural permeation in two methods. **(G)** The detection density of tumour necrosis in two methods. **(H)** The detection density of muscle invasion in two methods. **(I)** The detection rate of two methods in the four histological parameters. **(J)** The detection density of two methods in the four histological parameters.

The rate of detecting perineural permeation was significantly higher in the MSTS than RS specimens [88.46% (23/26 cases) vs. 65.38% (17/26 cases); *P* < 0.05]. Moreover, the detection density of perineural permeation was higher in the MSTS than RS specimens, although this difference did not reach statistical significance (2.00 vs. 1.07 times/cm^2^; *P* > 0.05). Similarly, the detection rate of tumour necrosis was higher, albeit not significantly, in the MSTS than RS specimens [84.62% (22/26 cases) vs. 61.54% (16/26 cases); *P* > 0.05]. The detection density of tumour necrosis was significantly higher in the MSTS than RS specimens (0.86 vs. 0.40 times/cm^2^; *P* < 0.01). The detection rate of muscle invasion was higher in MSTS than RS specimens, although this difference was not significant [92.31% (24/26 cases) vs. 76.92% (20/26 cases); *P* > 0.05]. MSTS resulted in a significantly higher detection density of muscle invasion than RS (1.14 vs. 0.51 times/cm^2^; *P* < 0.01; Figure 3).

### IHC Characteristics

RS resulted in a higher p53 detection rate than did MSTS, although the difference between the sampling methods was not significant [57.69% (15/26 cases) vs. 53.85%, (14/26 cases); *P* > 0.05]. Similarly, the average rate of p53 (mutant) positivity was higher, albeit not significantly, in the RS than MSTS specimens (74.32 vs. 69.67%; *P* > 0.05). The two sampling methods yielded the same p53 (mutant) expression status (positive or negative) in 25 of the 26 tumours. Among the 14 p53-positive MSTS specimens, 13 (92.86%) showed a statistically significant difference in the average positivity rate among the multi-site sampling blocks (*P* < 0.05).

Although not statistically significant, the average rate of Ki67 positivity was higher in the RS than MSTS specimens (58.15 vs. 58.06%; *P* > 0.05). Among the multi-site sampling blocks, the average positivity rate differed significantly (*P* < 0.05) in 17 (65.38%) tumours, whereas a significant trend (*P* = 0.05) was observed in 1 (3.85%) tumour. No statistically significant differences (*P* > 0.05) were observed in the remaining 8 (30.77%) tumours.

**Figure 4:**
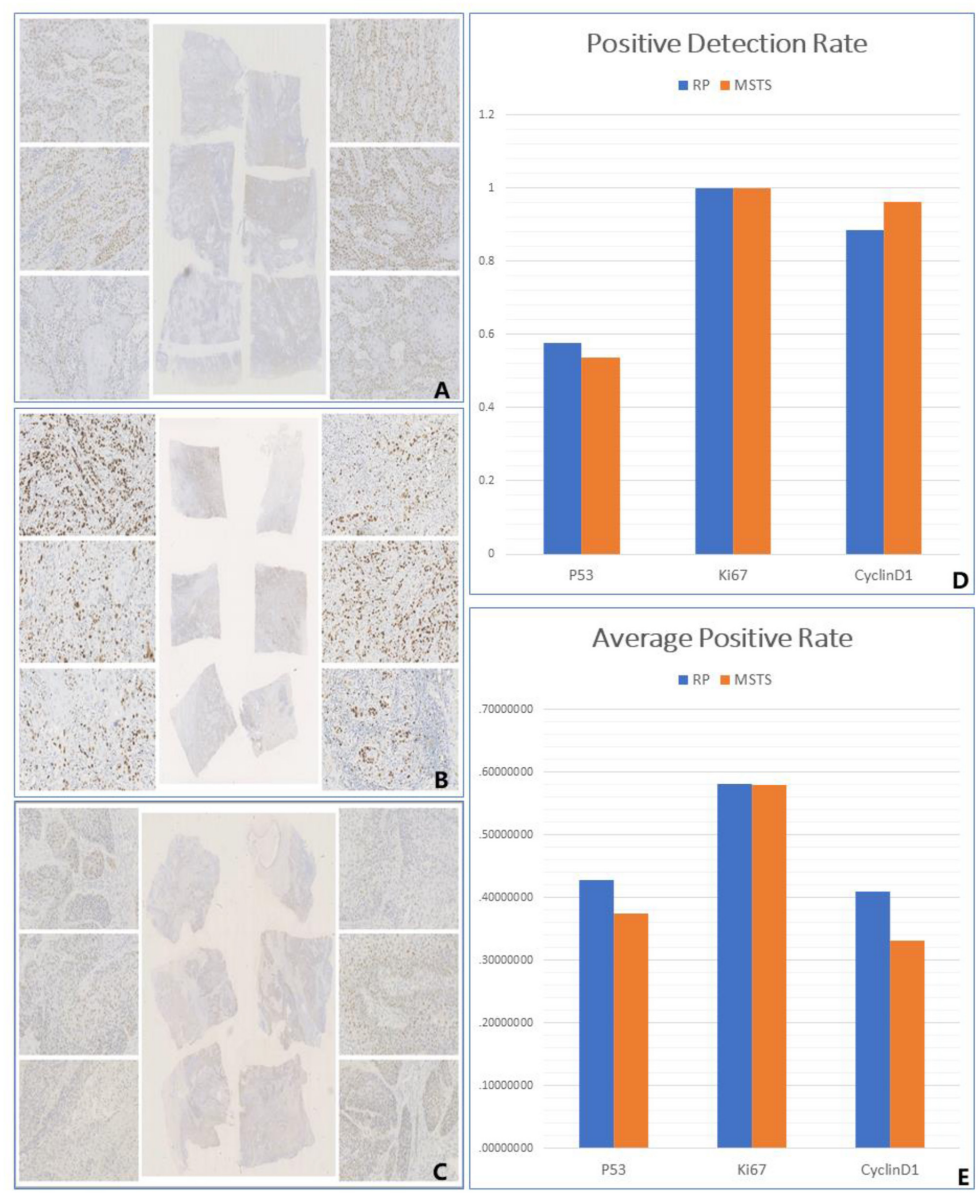
Immunohistology. **(A)** The differential expression of P53 among different small sampling blocks in the same multi-site sampling block. **(B)** The differential expression of Ki67 among different small sampling blocks in the same multi-site sampling block. **(C)** The differential expression of CyclinD1 among different small sampling blocks in the same multi-site sampling block. **(D)** The positive detection rate of two methods. **(E)** The average positive rate of two methods.

The detection rates of cyclin D1 expression in MSTS and RS specimens were 96.15% (25/26) and 88.46% (23/26), respectively. MSTS resulted in a higher detection rate of cyclin D1 expression than did RS, although the difference was not significant (*P* > 0.05). The average positivity rate of cyclin D1 was higher, albeit not significantly (*P* > 0.05), in the RS (46.27%) than MSTS (34.52%) specimens. The two sampling methods yielded the same cyclin D1 expression status (positive or negative) in 24 of the 26 tumours. Among 25 cyclin D1-positive MSTS specimens, 19 (76%) showed a significant difference in the average positivity rate among the multi-site sampling blocks (*P* < 0.05), 1 (4%) a significant trend (*P* = 0.05), and 5 (20%) no significant differences (*P* > 0.05; Figure 4).

### Mutation Detection

**Figure 5:**
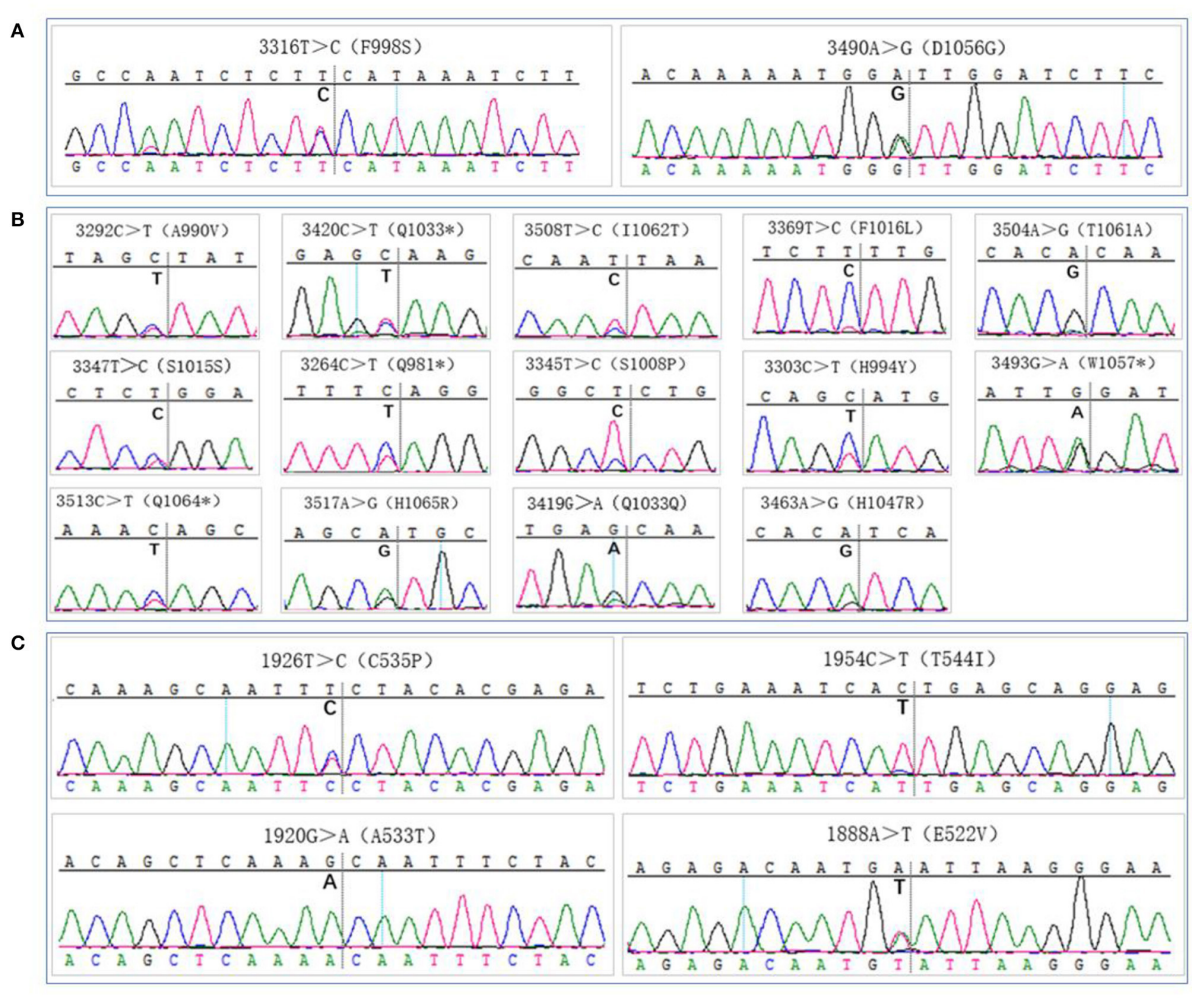
Gene mutation analysis. **(A)** PIK3CA gene mutation sites detection by routine sampling (focused on the exon 20), **(B,C)** PIK3CA gene mutation sites detection by multi-site tumour sampling [dispersed in exon 20 **(B)** and exon 9 **(C)**].

All samples were sequenced to detect mutations in PIK3CA exons 9 and 20, which are mutation hotspots. A total of 18 mutation sites were detected in eight MSTS specimens; however, 2 mutation sites were detected in only one RS specimen (case 13), which were also detected in the respective MSTS specimen (case 13M1). Hence, the mutation detection rate was significantly higher in the MSTS than RS specimens [40% (8/20) vs. 5% (1/20); *P* < 0.05]. Mutations in both exons were detected in the MSTS specimens, whereas mutations in only exon 20 were detected in the RS specimens. Two multi-site sampling blocks from the same patient were found to have the same mutation (3264C > T; cases: 17M2 and 17M4). The hotspot mutation H1047R, which has been shown to enhance PI3K lipid kinase activity, was detected in one MSTS specimen (case 24M5) but in no RS specimens. We also detected two mutations that did not affect the amino acid sequence (3347T > C and 3419G > A; Table 3, Figure 5).

**Table 3:** Detection of PIK3CA gene mutation of two methods.

**Number^①^**	**Sex/Age**	**Exon**	**Nucleotide definition^②^**	**Amino acid definition^③^**
13	F/72	20	3316T > C	F998S
13	F/72	20	3490A > G	D1056G
7M1	M/63	20	3292C > T	A990V
11M5	M/60	20	3420C > T	Q1033^*^
12M1	M/60	9	1926T > C	C535P
12M1	M/60	20	3508T > C	I1062T
12M2	M/60	20	3369T > C	F1016L
12M2	M/60	20	3504A > G	T1061A
13M1	F/72	20	3347T > C	S1015S
17M2	F/66	20	3264C > T	Q981^*^
17M4	F/66	20	3264C > T	Q981^*^
17M4	F/66	20	3345T > C	S1008P
17M5	F/66	20	3303C > T	H994Y
17M6	F/66	20	3493G > A	W1057^*^
19M1	F/45	20	3513C > T	Q1064^*^
19M3	F/45	20	3517A > G	H1065R
20M1	M/51	20	3419G > A	Q1033Q
23M2	M/54	9	1954C > T	T544I
23M5	M/54	9	1920G > A	A533T
24M4	M/67	9	1888A > T	E522V
24M5	M/67	20	3463A > G	H1047R

① *The number with “M” indicated the multi-site sampling block*.

② *mRNA NCBI reference sequence version: NM_006218.4*.

③ *Protein NCBI reference sequence version: NP_776999.1*.

* *Indicates protein translation ceased*.

### Methylation Detection

**Figure 6:**
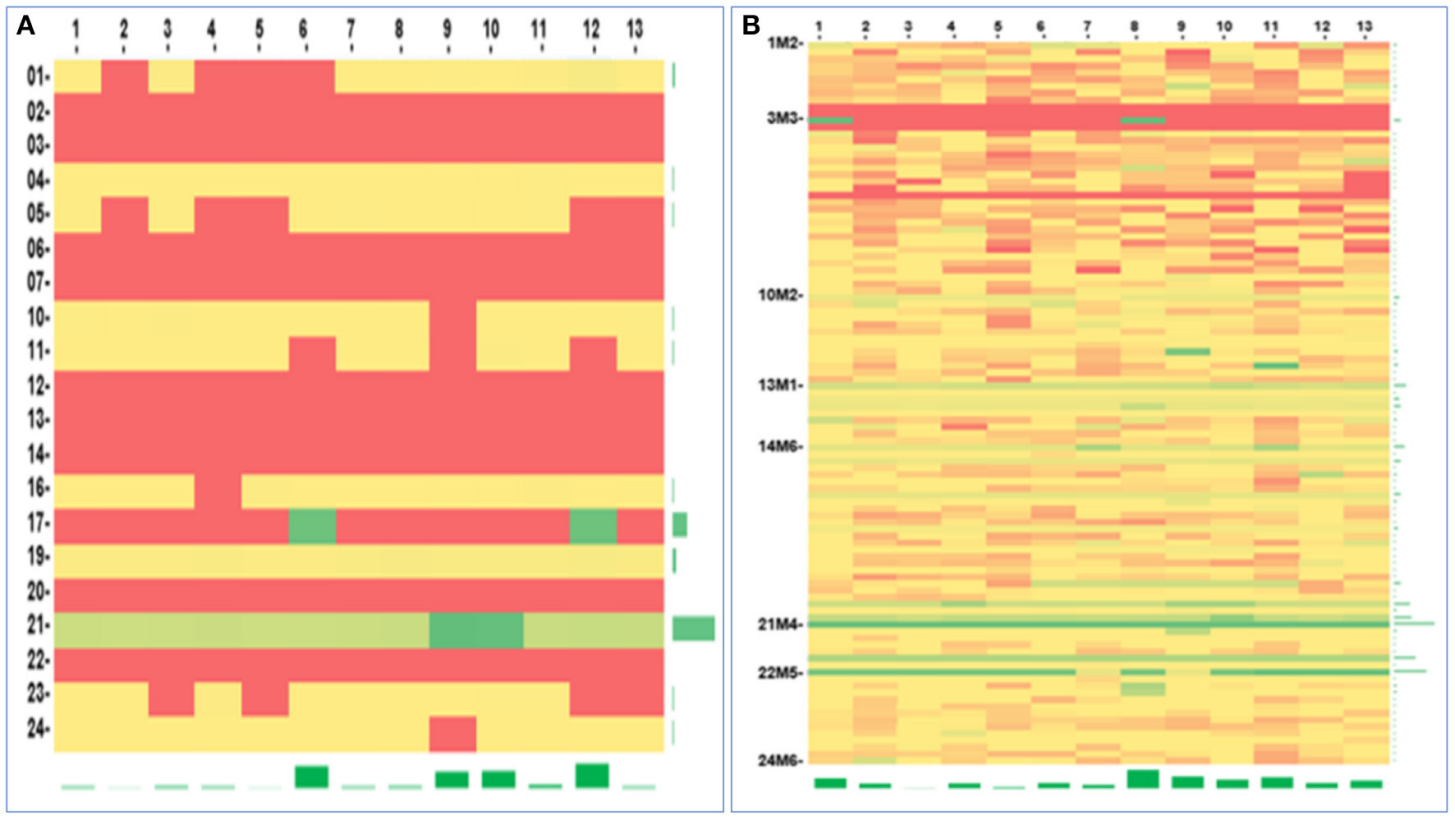
Gene methylation analysis. The heatmap of the CDKN2A gene promotor methylation detected by two methods: **(A)** Routine sampling (methylation rate: 0–53.68%), **(B)** Multi-site tumour sampling (methylation rate: 0–100%). The horizontal axis described the CpG sites (The number 1–13 were corresponding to the aligned gene sequence sites of 1936, 1941, 1943, 1947, 1951, 1954, 1960, 1982, 1986, 1989, 2007, 2010, 2013, respectively), and the vertical axis described the samples. Red, yellow, and green represent low, medium, and high methylation rates, respectively.

Thirteen CpG sites in the promoter region of the CDKN2A gene were analysed by next-generation sequencing. DNA methylation at these sites was detected in all 20 MSTS specimens tested. Nevertheless, only 11 RS specimens were found to have this methylation pattern; these methylation sites were also detected in the MSTS specimens. Therefore, the detection rate of DNA methylation was significantly higher in the MSTS than RS specimens [100% (20/20) vs. 55% (11/20); *P* < 0.01; Figure 6].

## Discussion

Lopez et al. developed an MSTS sampling method and demonstrated that it was an efficient and economical method to detect ITH in CCRCC (16). They also compared the Fuhrman grade, cell type (clear vs. granular eosinophilic), and tissue necrosis between samples obtained by MSTS and RS from 38 CCRCC patients. Importantly, MSTS yielded significantly higher rates of detecting high-grade and granular eosinophilic cells compared with RS (18). They also analysed PD-L1 (SP142) expression in 22 paired MSTS-obtained and RS-obtained CCRCC specimens and found a higher PD-L1 detection rate with MSTS than RS. Furthermore, MSTS revealed variations in PD-L1 expression within the same tumour (20) Consistent with these findings, the rates of detecting histological and IHC variations in OSCC and OPSCC tumours were higher in MSTS than RS specimens.

In this study, we assessed the ability of MSTS to detect ITH in various prognostic indicators, including peri-tumoural vascular and lymphatic growth, perineural permeation, tissue necrosis and muscle invasion. We found a superior performance of MSTS in detecting ITH in all these indicators compared with conventional sampling methods. These results suggested that MSTS improved the accuracy of pathological analysis of tumour samples, and could provide more information of tumour prognosis.

ITH in peri-tumoural vascular and lymphatic growth were more evident in the MSTS than RS specimens. Batsakis (24) first described tumour cell infiltration in peripheral nerves. Subsequently, Liebig et al. (25) proposed that tumour cells often infiltrate the nerve sheath. Perineural permeation is predictive of a poor prognosis and has been associated with lymph node involvement, metastasis, local recurrence, and tumour aggression (26–29). Here, we found a significantly higher rate of perineural permeation detection by MSTS than RS, rendering MSTS particularly useful for the detection of multifocal perineural permeation. Hasmat et al. (30) showed that multifocal perineural invasion was associated with a poor prognosis and higher mortality rate. Park et al. (29) confirmed that multifocal perineural invasion was associated with worse disease-free survival and disease-specific survival. Therefore, by improving the detection rate of multifocal perineural permeation, MSTS may improve patient risk stratification and treatment planning.

Rapid tumour proliferation and insufficient blood supply may enhance tumour necrosis, which has been identified as a predictor of poor prognosis in various solid malignancies, including breast cancer, rectal cancer, lung cancer, renal cell carcinoma, and advanced OSCC (31, 32). We found a higher rate of tumour necrosis detection with MSTS than RS.

Chandler et al. (33) proposed that muscle invasion can be used as a surrogate of invasion depth to assess lymph node involvement and the risk of local recurrence. Importantly, our results suggest that MSTS is superior to RS in terms of detecting muscle invasion in OSCC and OPSCC.

We analysed the expression of p53, Ki67, and cyclin D1 and detected regional expression patterns in the MSTS specimens that were not seen in the RS specimens. p53 is a critical tumour suppressor that maintains genetic stability. Mutations in p53 are important drivers of OSCC development (34) and are found in ~70% of OSCCs (35). p53 dysregulation promotes tumour growth, local invasion, and metastasis (36, 37). Interestingly, Khan et al. (38) reported that p53 upregulation was associated with poor prognosis in patients with advanced OSCC. Although we found no significant differences in the rate of p53 (mutant) positivity between the sampling methods, we detected a significant difference in the average positivity among multi-site blocks.

Ki67 is a widely used prognostic marker in numerous cancers, including breast, lung, prostate, and cervical cancers, as well as central nervous system malignancies (39). Evidence suggests that the Ki67 expression level predicts patient prognosis. Jing et al. (40) found that a high Ki67 expression level in the tumour core was associated with poor patient survival. Although we found no significant difference in the rate of Ki67 expression detection between MSTS and RS specimens, we observed differences in the average Ki67 positivity among multi-site blocks, indicating significant ITH in Ki67 expression.

Cyclin D1 is a crucial cell cycle regulator that promotes DNA synthesis and cell division by activating Cdk4 and Cdk6. Khan et al. (41) reported that the expression level of cyclin D1 in OSCC was associated with tumour size, lymph node involvement, clinical stage, as well as patient survival. High expression levels of cyclin D1 and p53 were associated with poor prognosis in patients with advanced OSCC, and co-expression of cyclin D1 and p53 was an independent prognostic factor for OSCC (38). Although we found no significant differences in the proportion of cyclin D1 positivity between the sampling methods, we detected a significant difference in the average positivity among multi-site blocks.

We also analysed the ability of MSTS to improve the detection rate of PIK3CA mutations and CDKN2A methylation. The PIK3CA gene is located on chromosome 3q26.3, and its mutation rate in OSCC ranges between 6 and 20% (42). Most PIK3CA mutations are found in exons 9 and 20, which encode the helical region and kinase region domain of PI3K, respectively (43). Here, we showed a significantly higher detection rate of PIK3CA mutations by MSTS than RS. In contrast to RS, which detected mutations only in exon 20, mutations in both exons were detected by MSTS. Importantly, the hotspot mutation H1047R was detected in the MSTS specimens. These results suggest that spatial variation exists in PIK3CA mutations, and that MSTS is superior to RS in detecting PIK3CA mutations. Methylation of tumour suppressor genes is frequent in cancer (44). Notably, CDKN2A promoter methylation is well-documented in head and neck malignancies (15). DNA promoter methylation would cause the silence of CDKN2A gene. CDKN2A regulates of cell division and apoptosis to take part in tumourigenesis, and decelerates cell cycle progression at G1/S phase to maintain the cellular homeostasis (45). CDKN2A silencing due to DNA methylation promotes cell proliferation and tumour development. CDKN2A gene methylation has been reported in 23–67% of primary OSCC tumours (15). In this study, we found a markedly higher detection rate of CDKN2A promoter methylation by MSTS than RS.

## Conclusion

We applied MSTS to prepare OPSCC specimens for the first time and found that MSTS was superior to RS in detecting ITH in OSCC and OPSCC, including variations in histology, gene expression, clinicopathological characteristics, and mutational and epigenetic landscapes.

## Data Availability Statement

The datasets used and analyzed during the current study may be made available by the corresponding author upon reasonable request.

## Ethics Statement

The studies involving human participants were reviewed and approved by the Ethics of Committee of the Peking University Hospital of Stomatology. The patients/participants provided their written informed consent to participate in this study.

## Author Contributions

B-bL initiated and designed the study and revised the manuscript. WJ did the experiments, collected and analysed the data, and drafted the manuscript. JB and JY did the experiments. YC collected the data. All authors approved the final version.

## Conflict of Interest

The authors declare that the research was conducted in the absence of any commercial or financial relationships that could be construed as a potential conflict of interest.
