# Supplementary material for: Multi-Site Tumour Sampling Improves the Detection of Intra-Tumour Heterogeneity in Oral and Oropharyngeal Squamous Cell Carcinoma
Source: Front Med (Lausanne). 2021 May 10;8:670305. doi: 10.3389/fmed.2021.670305 (PMC8141800; doi:10.3389/fmed.2021.670305)
Supplement: Supplementary file 1 [file Data_Sheet_1.zip › Supplementary Material/The raw data for figure5 and 6/Electropherogram for CDKN2A and PIK3CA.docx]

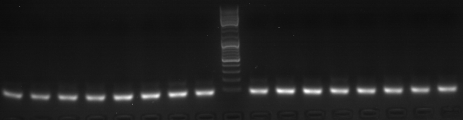


1 2 3 4 5 6 7 8 Marker 9 10 11 12 13 14 15 16


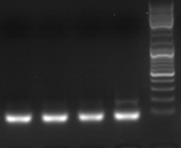

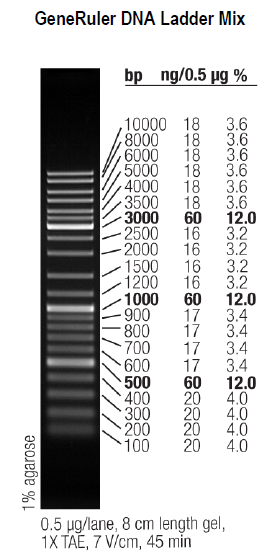


17 18 19 20 Marker

FIGURE 1 | Electropherogram for CDKN2A gene promotor (RP).


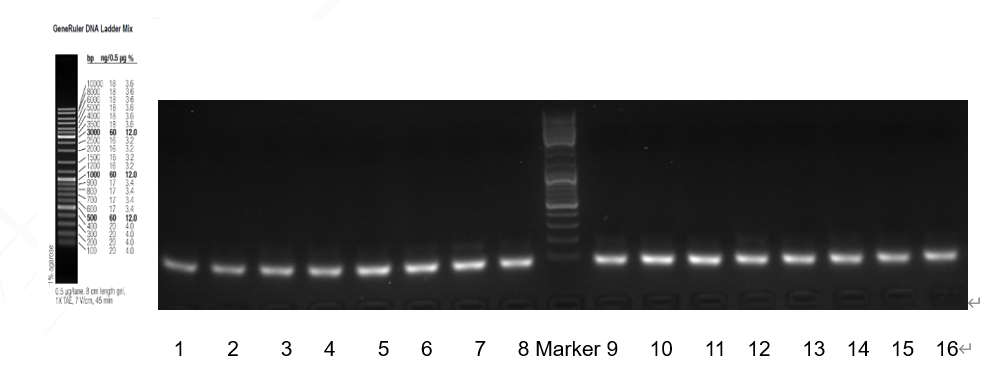

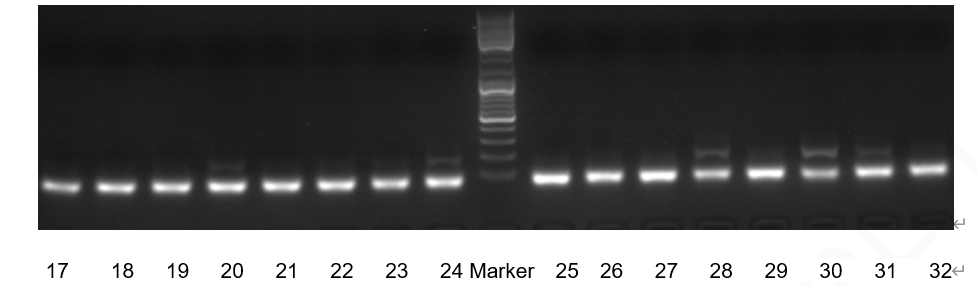

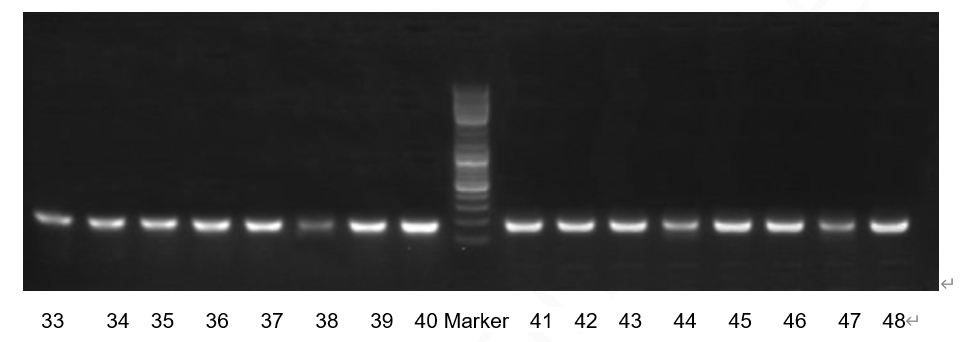

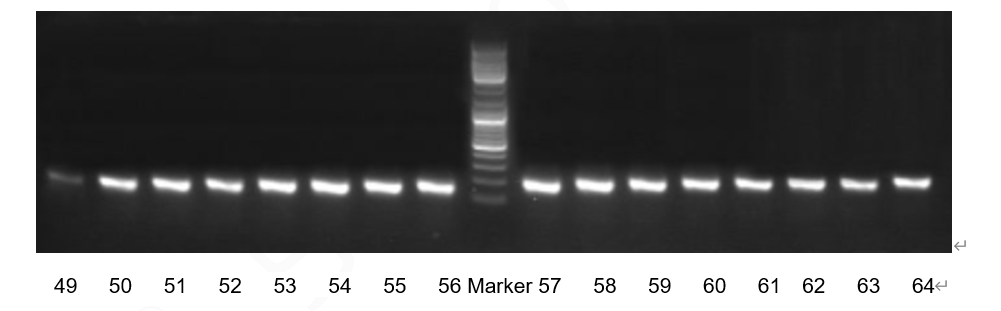

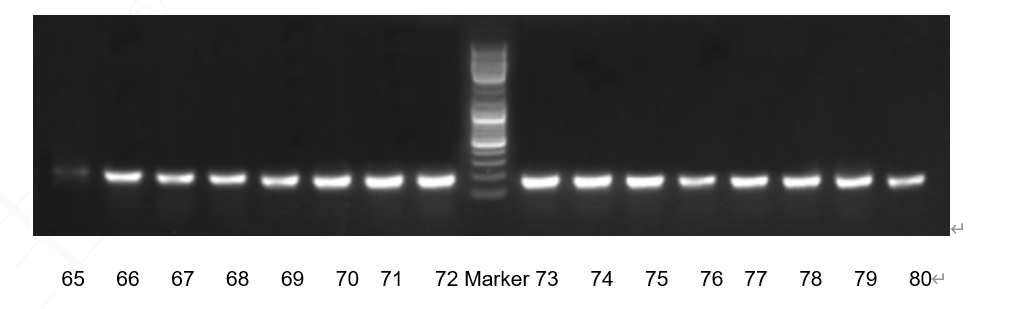

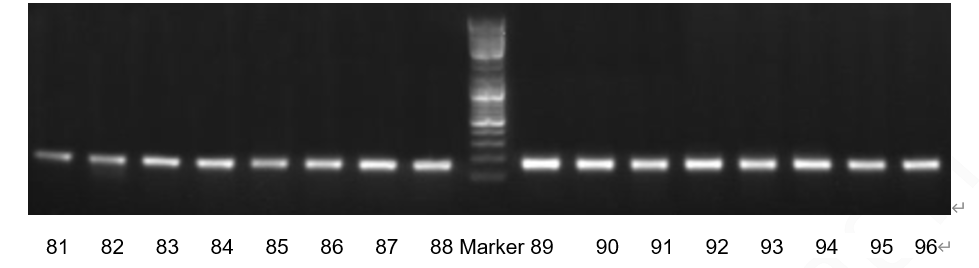

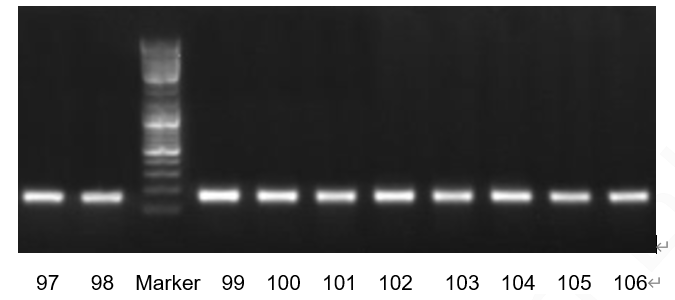


FIGURE 2 | Electropherogram for CDKN2A gene promotor (MSTS).


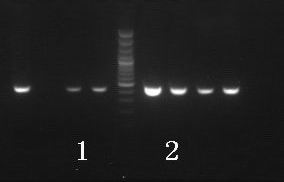


FIGURE 3 | Electropherogram for PIK3CA gene (1: exon 9; 2: exon 20).
