# Supplementary material for: Multi-Site Tumour Sampling Improves the Detection of Intra-Tumour Heterogeneity in Oral and Oropharyngeal Squamous Cell Carcinoma
Source: Front Med (Lausanne). 2021 May 10;8:670305. doi: 10.3389/fmed.2021.670305 (PMC8141800; doi:10.3389/fmed.2021.670305)

100-3000bp Ladder-K

Cat. No. : B500347
Package: 50 loads
Storage: Store at -20°C
Brand: 生工
Ready to use

Description

100-3000 bp Ladder-K, ready-to-use, contains a mix of 14 individual DNA fragments (in base pairs): 100, 200, 300, 400, 500, 600, 700, 800, 900, 1000, 1200, 1500, 2000, 3000 bp.

Usage

6 µl, (containing 50 ng DNA each band)

Storage Buffer

10 mM Tris-HCl (pH 7.6), 10 mM EDTA, 0.033% Bromophenol Blue, 0.008% xylene cyanol FF and 10% glycerol.

Quality Control Assay Data

Analysis of 0.5 µg of the DNA Ladder on agarose gel by ethidium bromide staining generates 10 discrete bands pattern.

Recommendation For Use

- Do not heat before loading.
- Apply 5 µl marker to 5 mm width lane (1.2 µl per 1 mm lane) agarose gel or non-denaturing PAGE.
- Following electrophoretic separation on gels, visualize the DNA bands by ethidium bromide staining.
- Not designed for DNA quantification.
- Not designed for denaturing PAGE.
- The 100 bp band will be faint after long term electrophoresis.
- To get desired photo you can visualize the DNA bands by ethidium bromide staining after electrophoresis.
- Qualified agarose and fresh TAE (TBE) buffer is essential to the good photo.

Product Use limitation

This Product is developed, designed and sold exclusively for research purposes and in vitro use only. The product was not tested for use in diagnostics or for drug development, nor is it suitable for administration to human or animal.

References

- Stellwagen, N.C., Anomalous electrophoresis of deoxyribonucleic acid restriction fragments on polyacrylamide gels, Biochemistry, 22, 6186-6193, 1983.
- Stellwagen, N.C., Conformational isomers of curved DNA molecules can be observed by polyacrylamide gel electrophoresis, Electrophoresis, 21, 2327-2334, 2000.

100-3000 bp Ladder-K

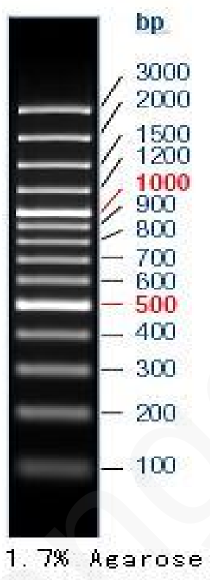

Supplement: Supplementary file 1 [file Data_Sheet_1.zip › Supplementary Material/The raw data for figure5 and 6/manual for ladder 1.pdf]
