# Supplementary material for: Multi-Site Tumour Sampling Improves the Detection of Intra-Tumour Heterogeneity in Oral and Oropharyngeal Squamous Cell Carcinoma
Source: Front Med (Lausanne). 2021 May 10;8:670305. doi: 10.3389/fmed.2021.670305 (PMC8141800; doi:10.3389/fmed.2021.670305)
Supplement: Supplementary file 1 [file Data_Sheet_1.zip › Supplementary Material/The raw data for figure5 and 6/manual for ladder 2.pdf]

thermo scientific

PRODUCT INFORMATION

Thermo Scientific GeneRuler DNA Ladder Mix

Pub. No. MAN0013012
Rev. Date 12 February 2018 (Rev. C.00)

Components	#SM0331	#SM0332
GeneRuler DNA Ladder Mix, 0.5 µg/µL	250 (5 x 50) µg (for 500 applications)	1250 (25 x 50) µg (for 2500 applications)
6X TriTrack DNA Loading Dye	2 × 1 mL	10 × 1 mL

Store at -25°C to -15°C

www.thermofisher.com
For Research Use Only. Not for use in diagnostic procedures.

Description

Thermo Scientific™ GeneRuler™ DNA Ladder Mix is designed for sizing and approximate quantification of wide range double-stranded DNA on agarose gel. The ladder is composed of 21 chromatography-purified DNA fragments (in base pairs): 10000, 8000, 6000, 5000, 4000, 3500, **3000**, 2500, 2000, 1500, 1200, **1000**, 900, 800, 700, 600, **500**, 400, 300, 200, and 100. It contains three reference bands (3000, 1000 and 500 bp) for easy orientation.
The ladder is dissolved in TE buffer.

Storage Buffer

10 mM Tris-HCl (pH 7.6), 1 mM EDTA.

6X TriTrack DNA Loading Dye

10 mM Tris-HCl (pH 7.6), 0.03% bromophenol blue, 0.03% xylene cyanol FF, 0.15% orange G, 60% glycerol and 60 mM EDTA.

Protocol for Loading

Loading mixture for the 5 mm agarose gel lane*:

DNA Ladder	1 µL
6X TriTrack DNA Loading Dye	1 µL
Deionized water	4 µL
	6 µL

Step 1: Mix gently

Step 2: Load on the gel

* For gels with other lane widths, the components of the mixture should be scaled either up or down. Use 0.2 µL (0.1 µg) of DNA Ladder per 1mm of lane.

Recommendations

- Do not heat before loading.
- Dilute your DNA sample with the 6X TriTrack DNA Loading Dye (#R1161, supplied with the ladder): mix 1 volume of the dye solution with 5 volumes of the DNA sample;
- Load the same volumes of the DNA sample and the DNA ladder;
- For quantification, adjust the concentration of the sample to equalize it approximately with the amount of DNA in the nearest band of the ladder.
- For DNA band visualization with SYBR™ Green and other intercalating dyes, do not add the dyes into the sample, use gel staining after electrophoresis or include dyes into agarose gel to avoid aberrant DNA migration.
- **Important note:** For DNA bands visualization with GelRed™ use gel staining after electrophoresis to avoid aberrant DNA migration.

GeneRuler DNA Ladder Mix

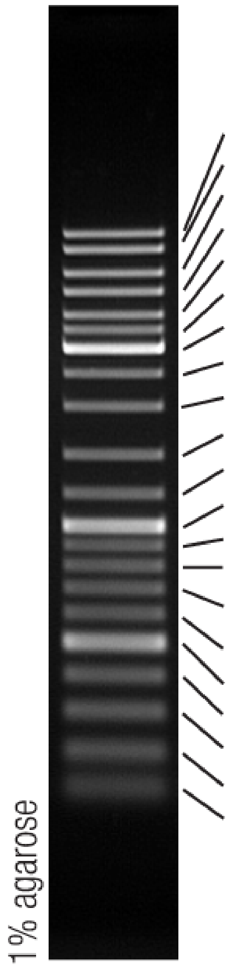

bp	ng/0.5 µg	%
10000	18	3.6
8000	18	3.6
6000	18	3.6
5000	18	3.6
4000	18	3.6
3500	18	3.6
**3000**	**60**	**12.0**
2500	16	3.2
2000	16	3.2
1500	16	3.2
1200	16	3.2
**1000**	**60**	**12.0**
900	17	3.4
800	17	3.4
700	17	3.4
600	17	3.4
**500**	**60**	**12.0**
400	20	4.0
300	20	4.0
200	20	4.0
100	20	4.0

0.5 µg/lane, 8 cm length gel,
1X TAE, 7 V/cm, 45 min

LIMITED USE LABEL LICENSE: Internal Research and Development Use Only.

The purchase of this product conveys to the buyer the limited, non-exclusive, non-transferable right (without the right to resell, repackage, or further sublicense) to use this product for internal research and development purposes. No other license is granted to the buyer whether expressly, by implication, by estoppel or otherwise. In particular, the purchase of the product does not include or carry any right or license to use, develop, or otherwise exploit this product commercially and no rights are conveyed to the buyer to use the product or components of the product for purposes including but not limited to provision of services to a third party, generation of commercial databases or clinical diagnostics. This product is sold pursuant to authorization from Thermo Fisher Scientific and Thermo Fisher Scientific reserves all other rights. For information on purchasing a license for uses other than internal research and development purposes, please contact outlicensing@lifetech.com or Out Licensing, Life Technologies Inc., 5781 Van Allen Way, Carlsbad, California 92008.

PRODUCT USE LIMITATION

This product is developed, designed and sold exclusively *for research purposes and in vitro use only*. The product was not tested for use in diagnostics or for drug development, nor is it suitable for administration to humans or animals.

Please refer to www.thermofisher.com for Material Safety Data Sheet of the product.

© 2018 Thermo Fisher Scientific Inc. All rights reserved. All trademarks are the property of Thermo Fisher Scientific Inc. and its subsidiaries.
