# Supplementary figures and images for: Multi-Site Tumour Sampling Improves the Detection of Intra-Tumour Heterogeneity in Oral and Oropharyngeal Squamous Cell Carcinoma
Source: Front Med (Lausanne). 2021 May 10;8:670305. doi: 10.3389/fmed.2021.670305 (PMC8141800; doi:10.3389/fmed.2021.670305)

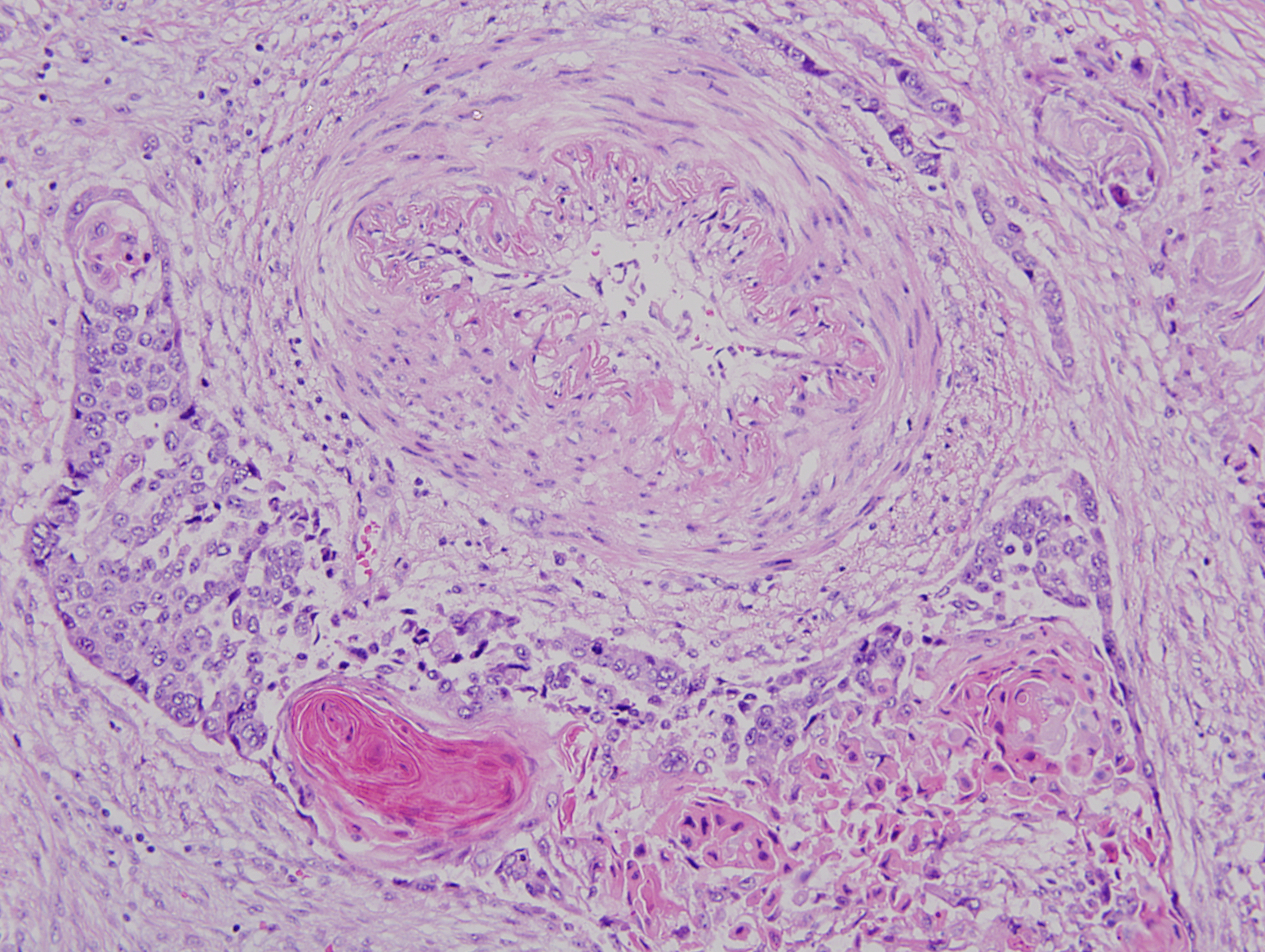

Supplement: Supplementary file 1 [file Data_Sheet_1.zip › Supplementary Material/The raw data for figure3/(A) Peritumoral vascular and lymphatic growth (20x).jpg]

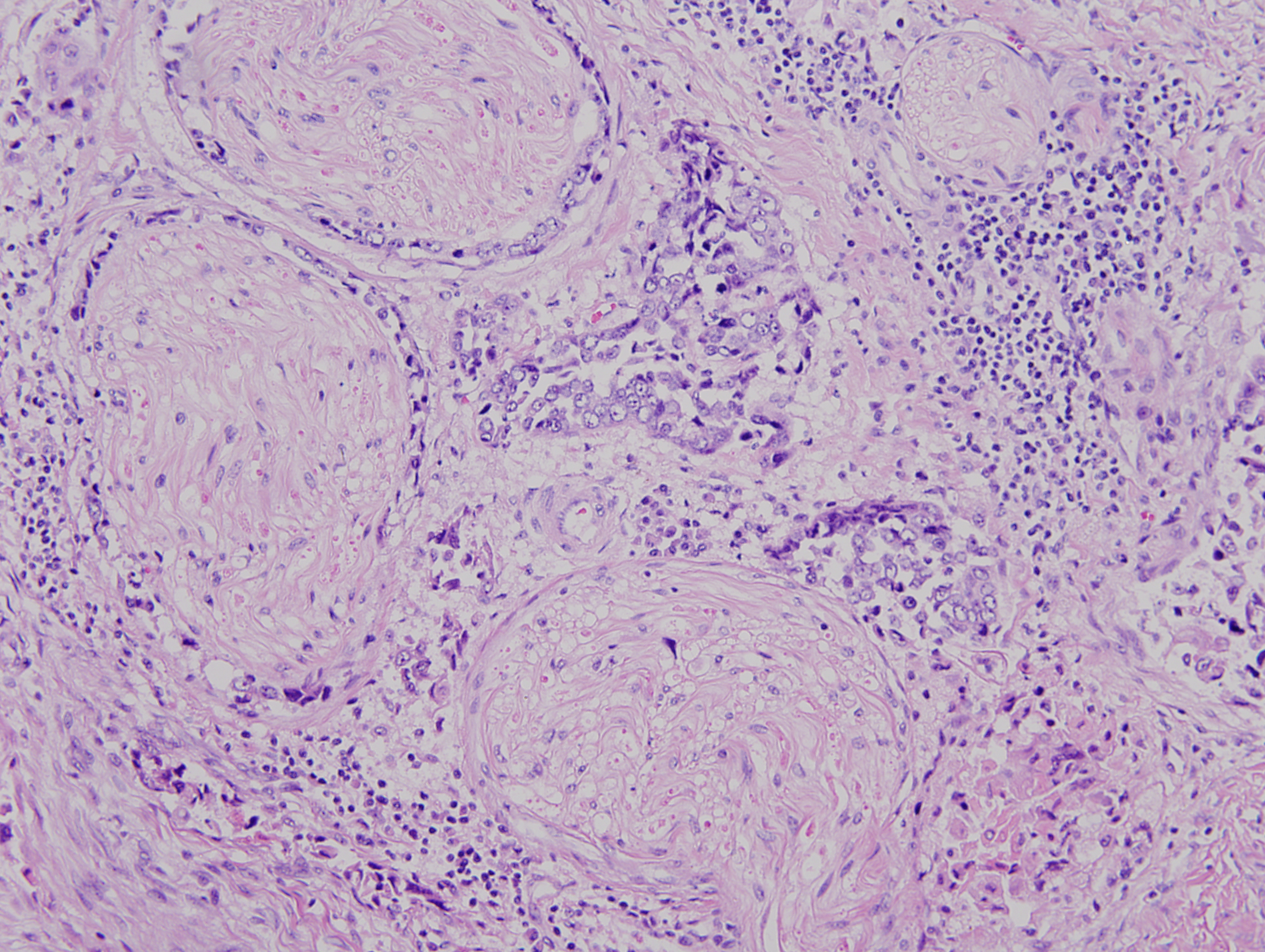

Supplement: Supplementary file 1 [file Data_Sheet_1.zip › Supplementary Material/The raw data for figure3/(B) Perineural permeation(20X).jpg]

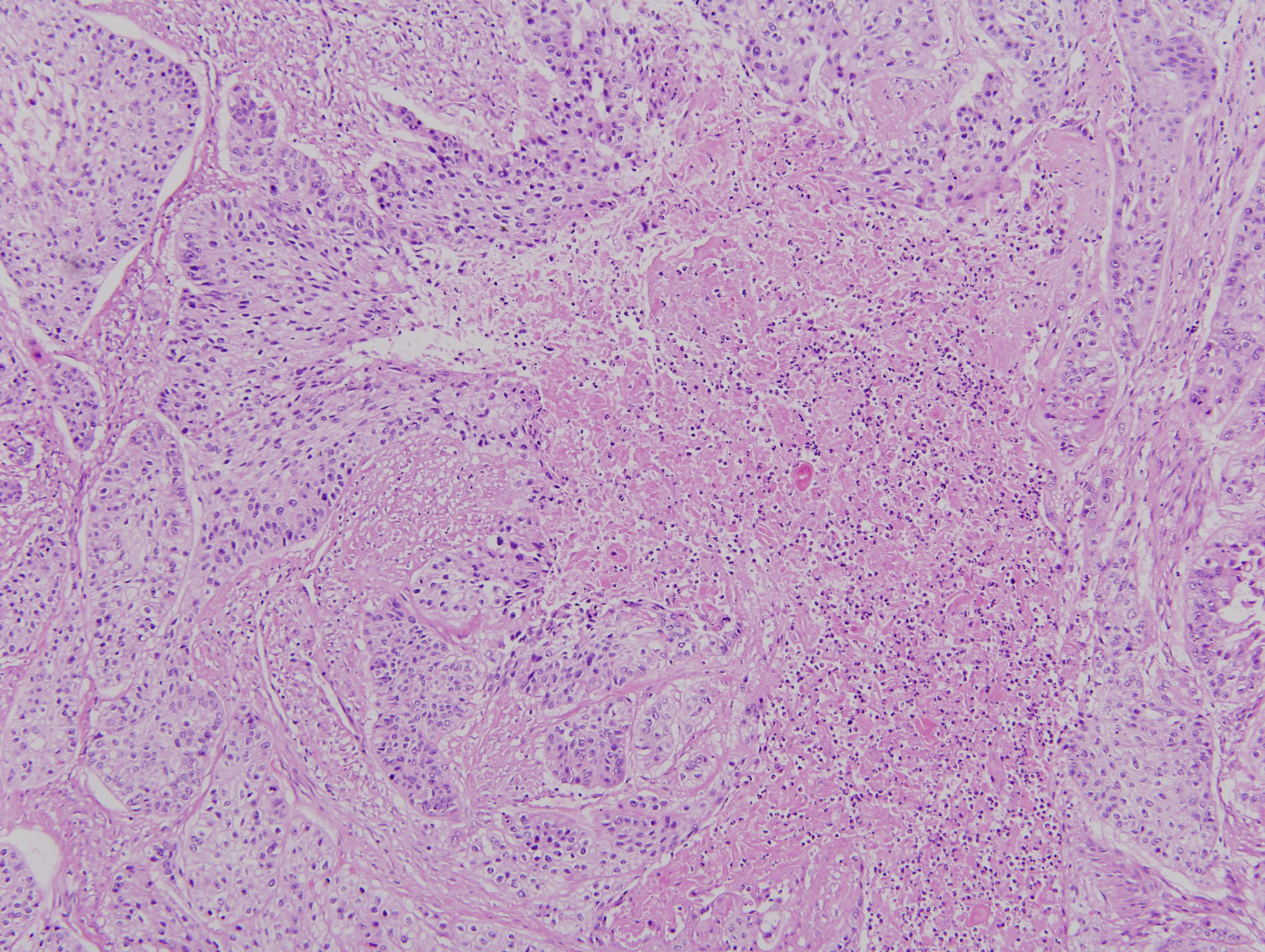

Supplement: Supplementary file 1 [file Data_Sheet_1.zip › Supplementary Material/The raw data for figure3/(C)Tumour necrosis(20x).jpg]

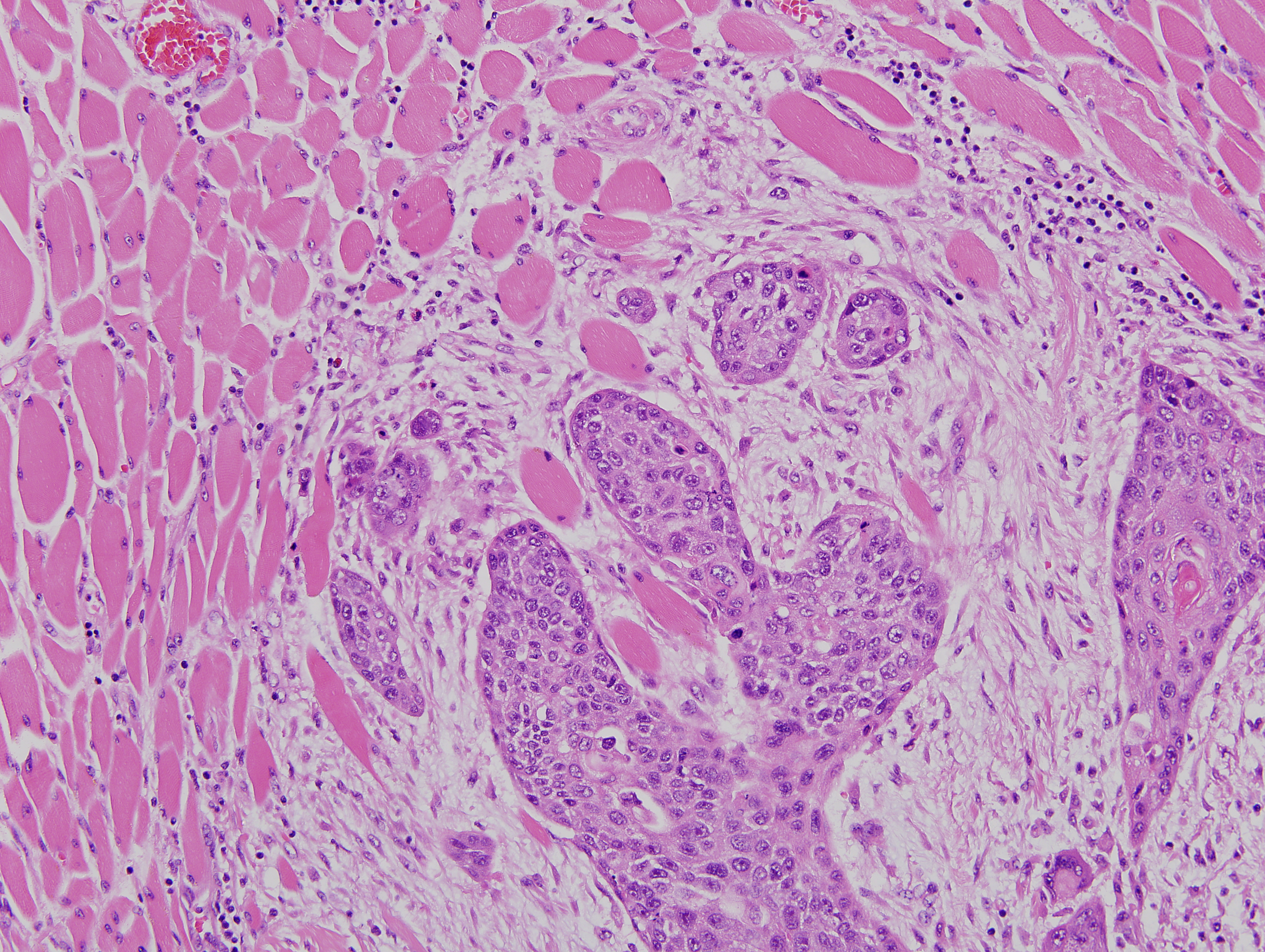

Supplement: Supplementary file 1 [file Data_Sheet_1.zip › Supplementary Material/The raw data for figure3/(D)Muscle invasion(20x).jpg]

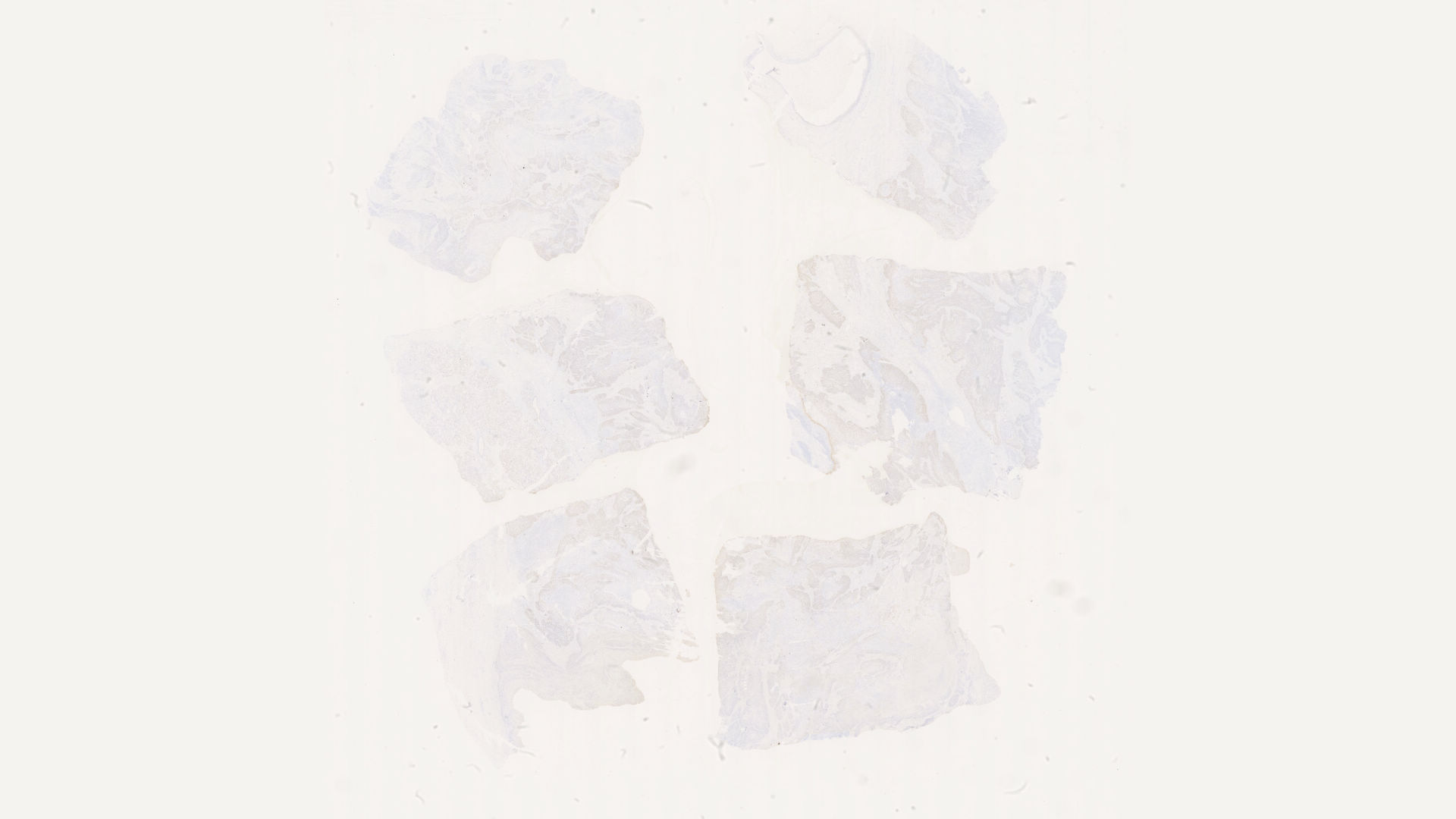

Supplement: Supplementary file 1 [file Data_Sheet_1.zip › Supplementary Material/The raw data for figure4/(C)The differential expression of CyclinD1 among different small sampling blocks in the same multi-site sampling block/FIGURE 4. (C)-center (0.45x).jpg]

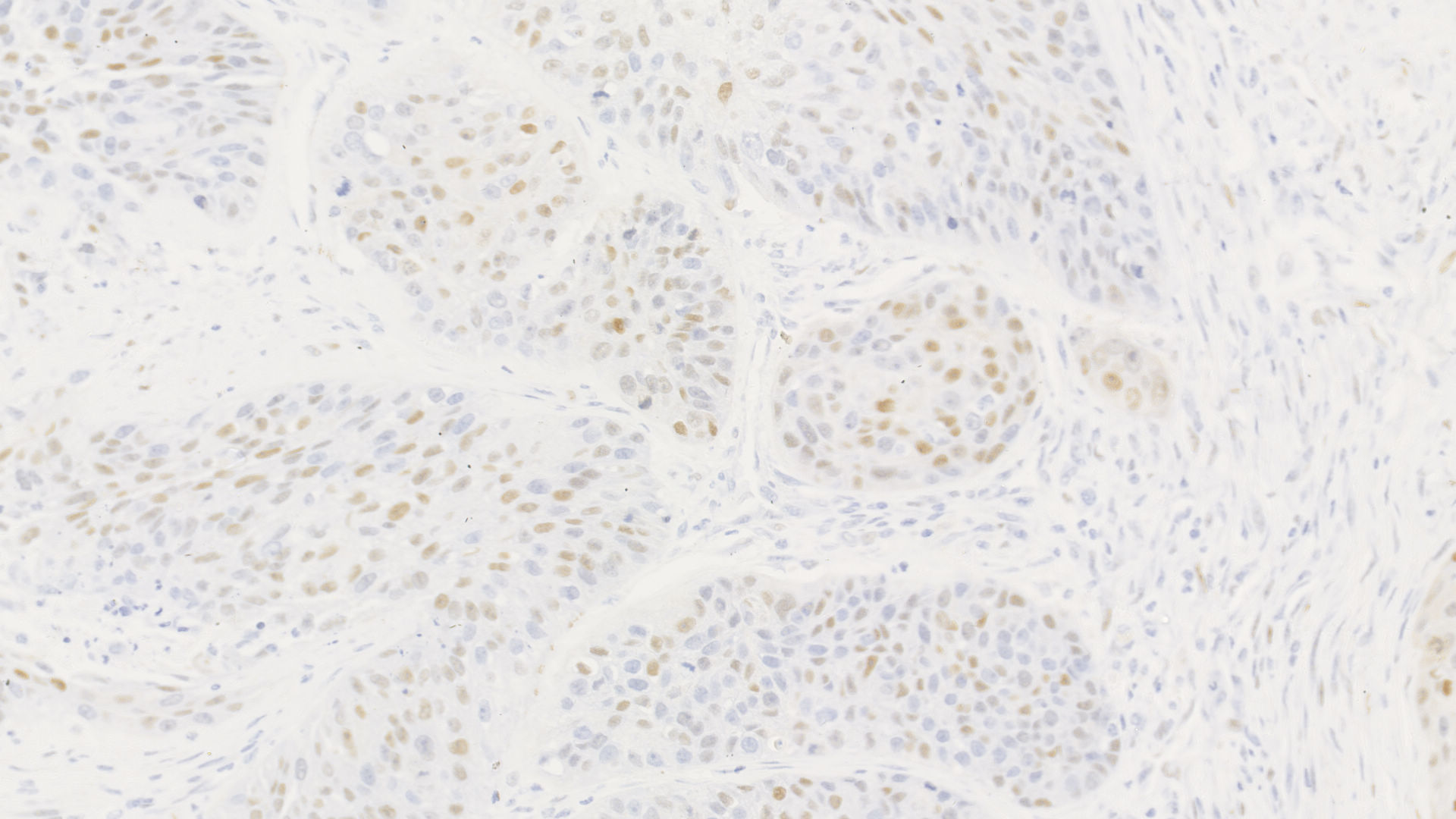

Supplement: Supplementary file 1 [file Data_Sheet_1.zip › Supplementary Material/The raw data for figure4/(C)The differential expression of CyclinD1 among different small sampling blocks in the same multi-site sampling block/FIGURE 4. (C)-left 1(20x).jpg]

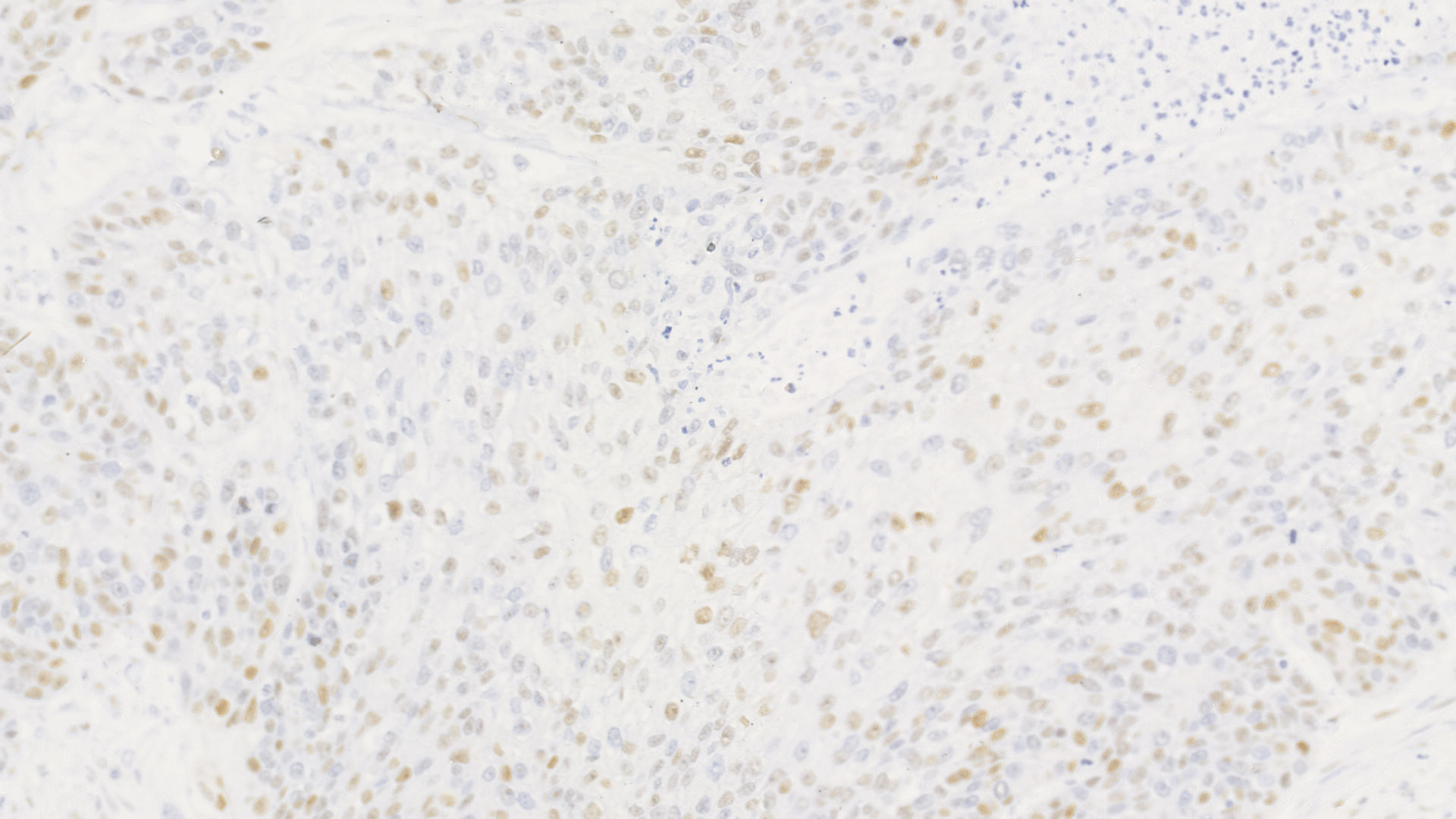

Supplement: Supplementary file 1 [file Data_Sheet_1.zip › Supplementary Material/The raw data for figure4/(C)The differential expression of CyclinD1 among different small sampling blocks in the same multi-site sampling block/FIGURE 4. (C)-left 2(20x).jpg]

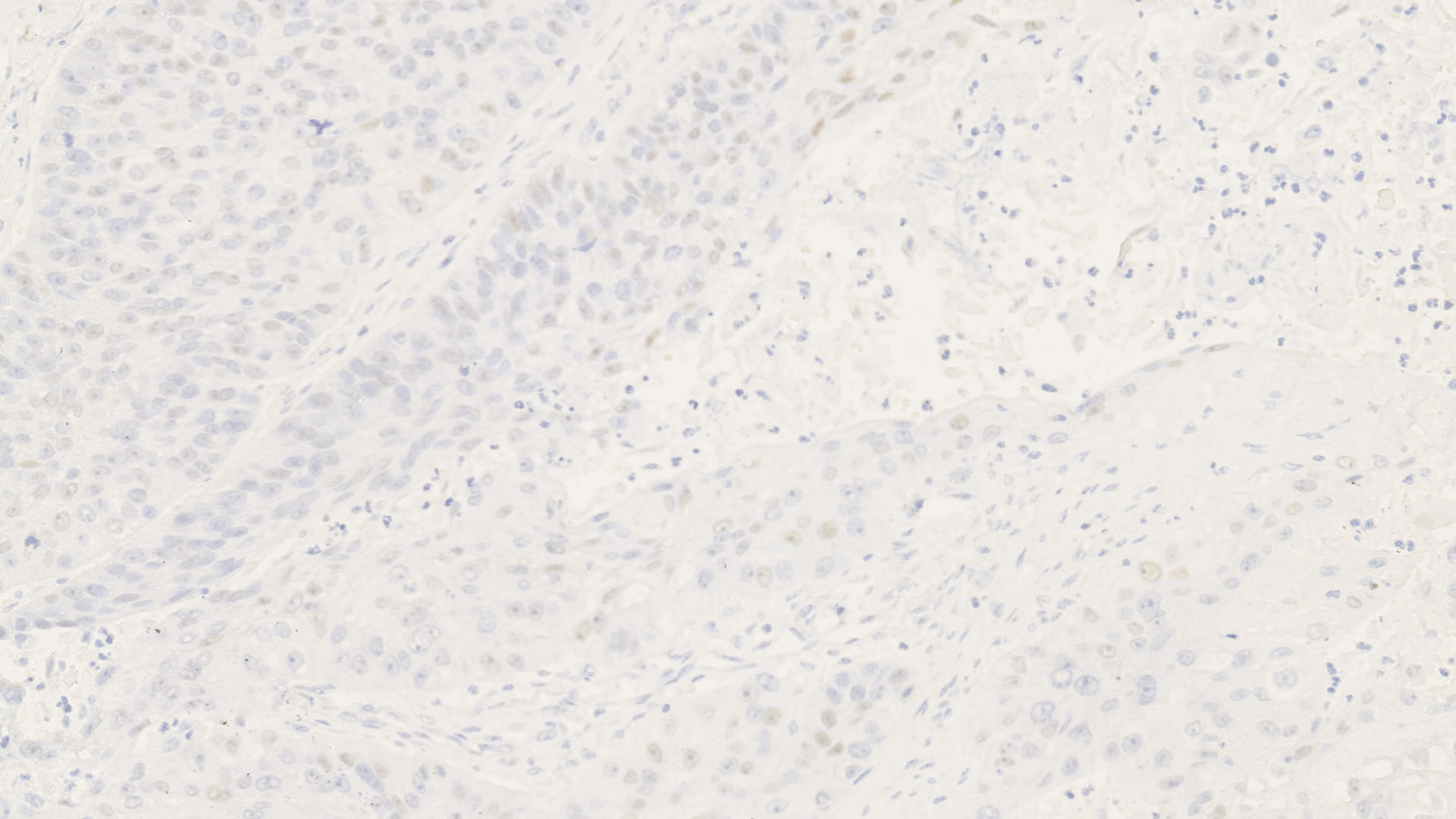

Supplement: Supplementary file 1 [file Data_Sheet_1.zip › Supplementary Material/The raw data for figure4/(C)The differential expression of CyclinD1 among different small sampling blocks in the same multi-site sampling block/FIGURE 4. (C)-left 3(20x).jpg]

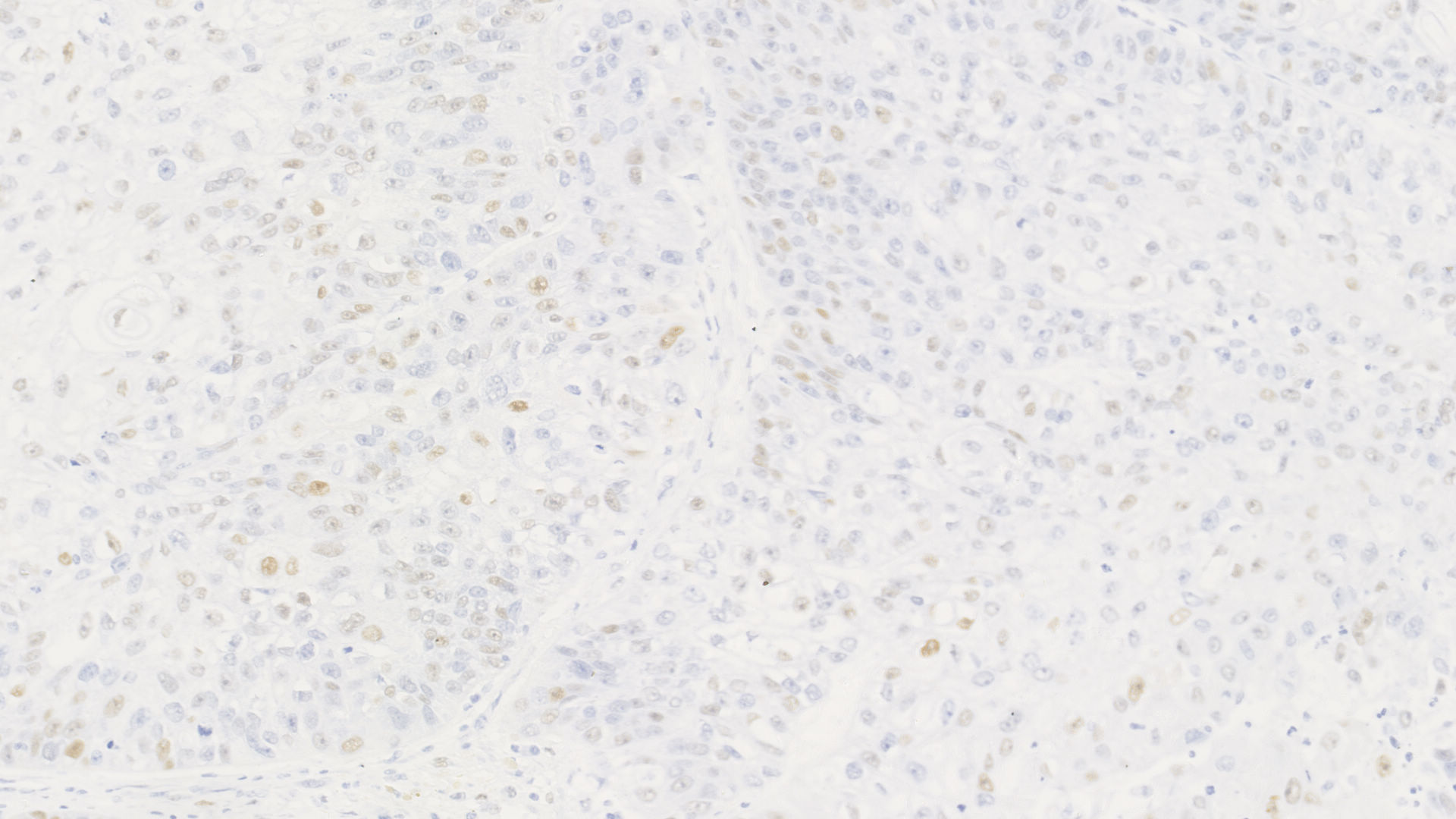

Supplement: Supplementary file 1 [file Data_Sheet_1.zip › Supplementary Material/The raw data for figure4/(C)The differential expression of CyclinD1 among different small sampling blocks in the same multi-site sampling block/FIGURE 4. (C)-right 1(20x).jpg]

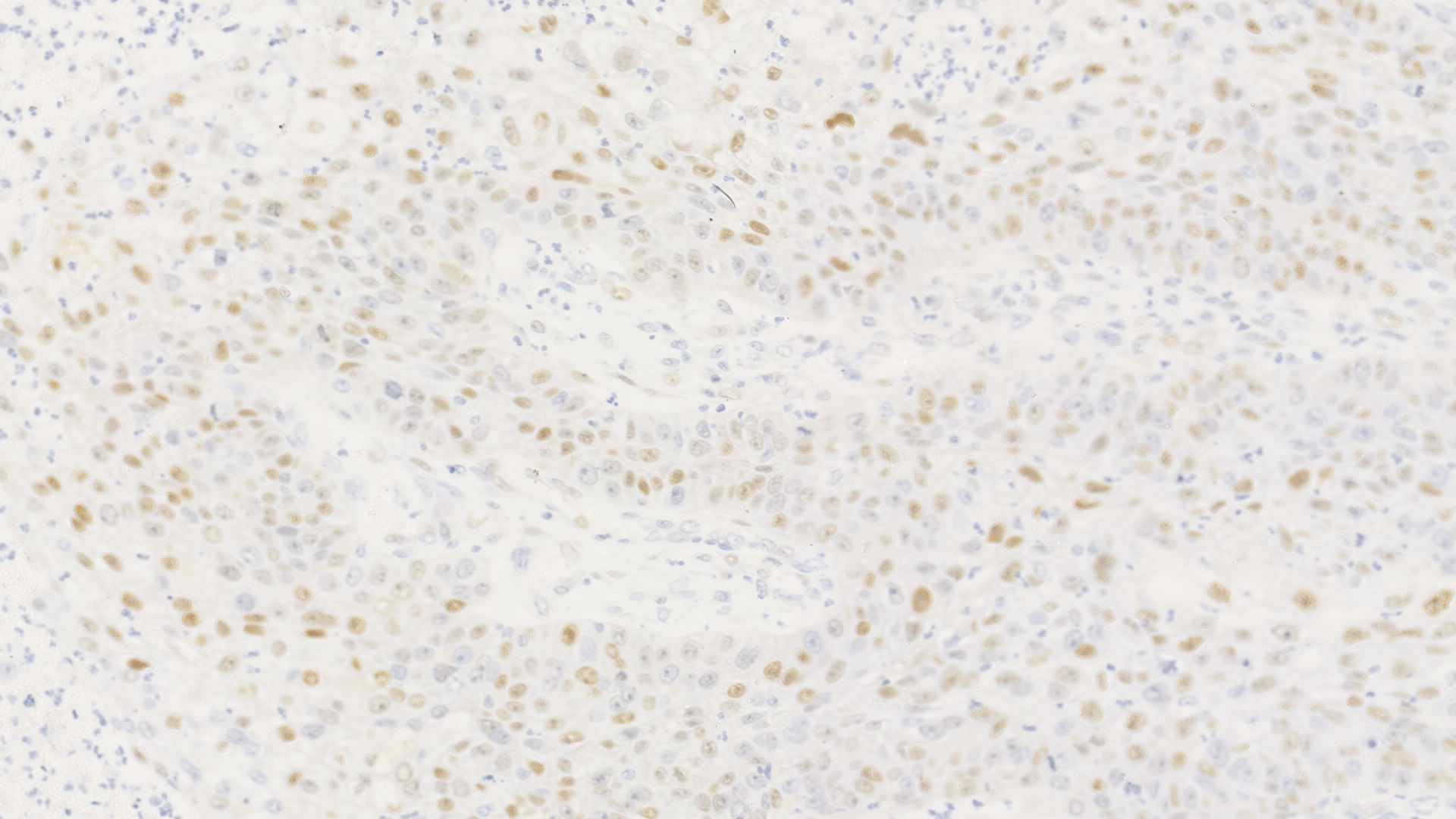

Supplement: Supplementary file 1 [file Data_Sheet_1.zip › Supplementary Material/The raw data for figure4/(C)The differential expression of CyclinD1 among different small sampling blocks in the same multi-site sampling block/FIGURE 4. (C)-right 2(20x).jpg]

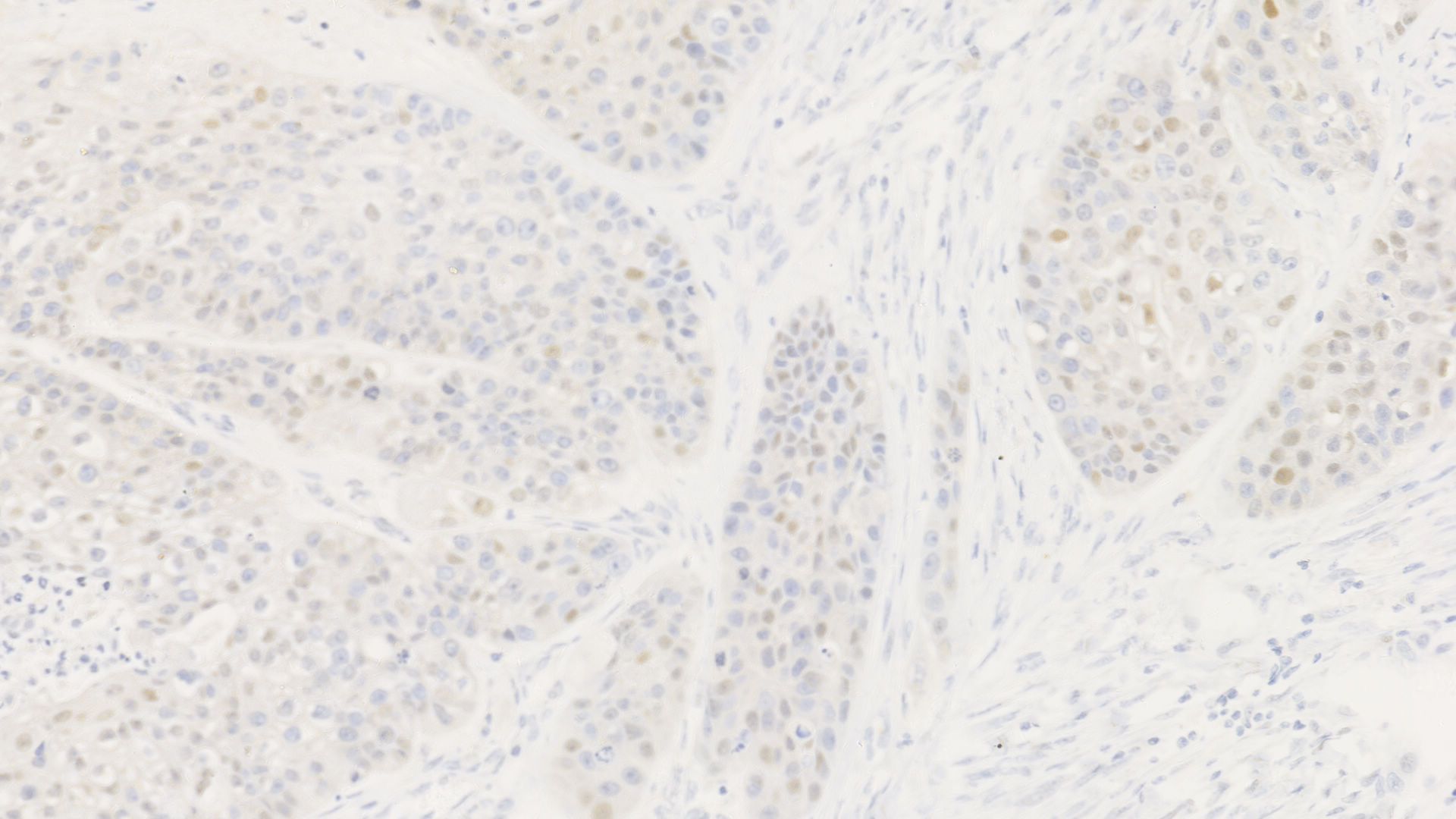

Supplement: Supplementary file 1 [file Data_Sheet_1.zip › Supplementary Material/The raw data for figure4/(C)The differential expression of CyclinD1 among different small sampling blocks in the same multi-site sampling block/FIGURE 4. (C)-right3(20x).jpg]

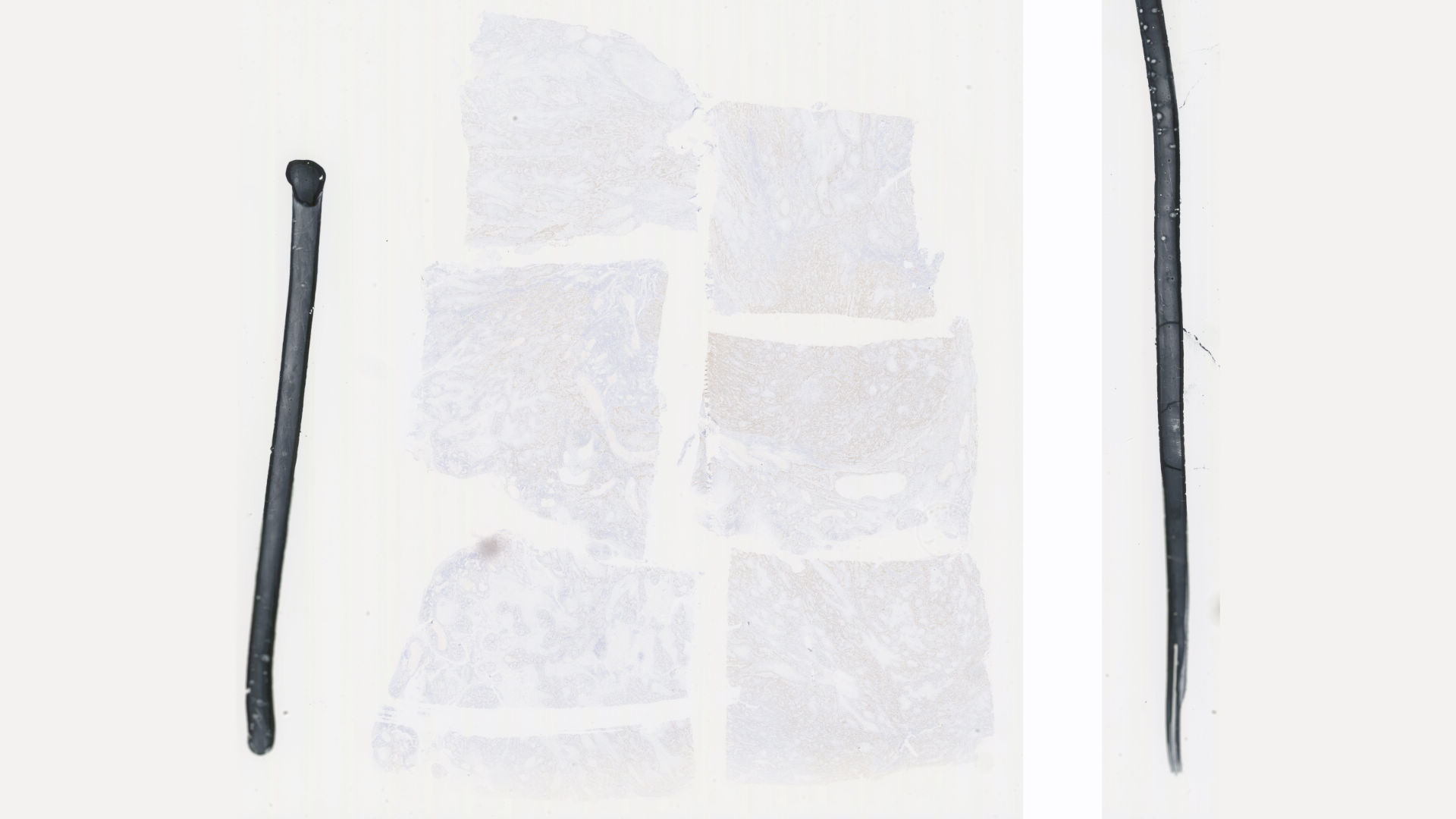

Supplement: Supplementary file 1 [file Data_Sheet_1.zip › Supplementary Material/The raw data for figure4/ú¿Aú⌐The differential expression of P53 among different small sampling blocks in the same multi-site sampling block/FIGURE 4. (A)-center(0.51x).jpg]

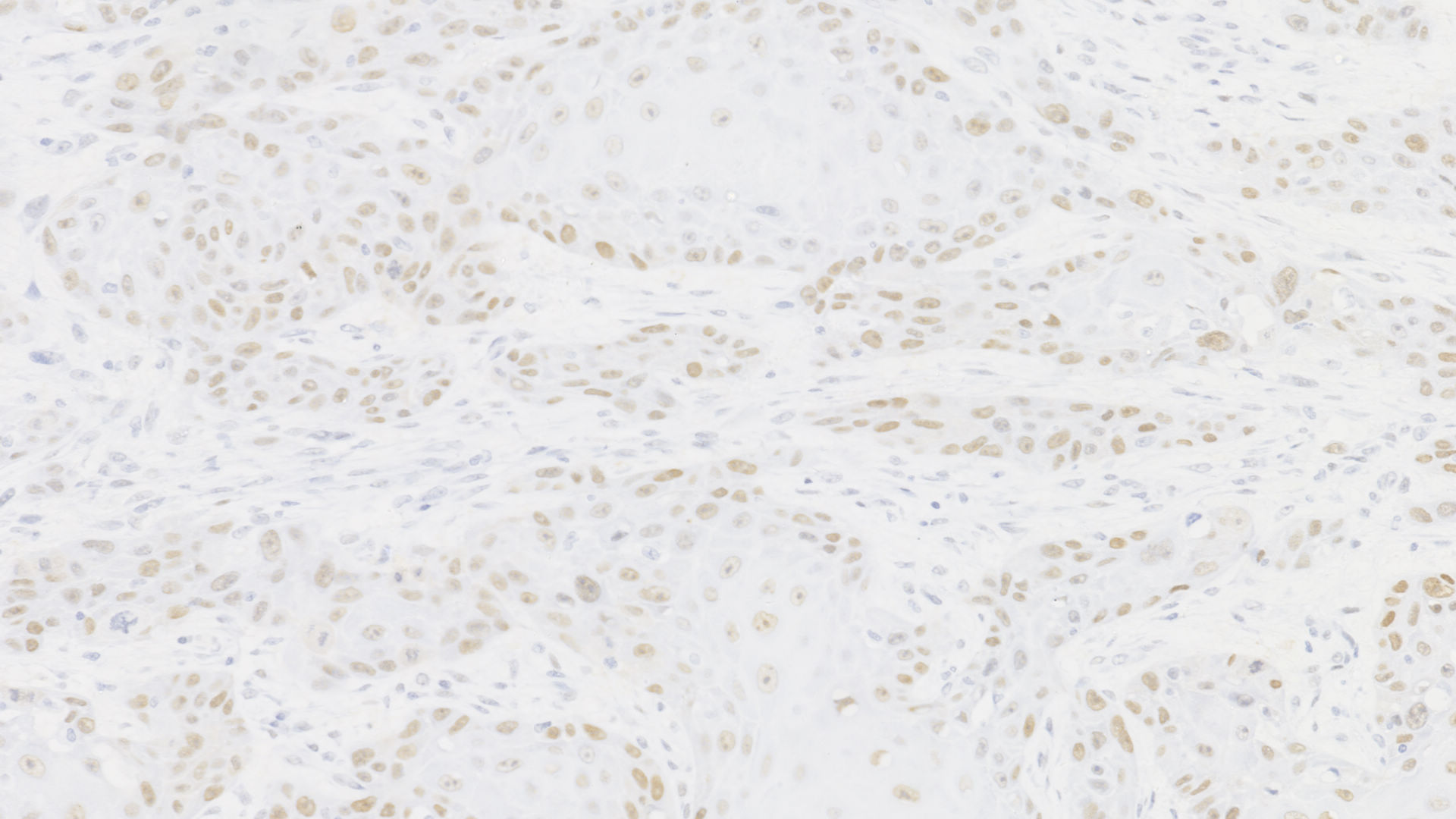

Supplement: Supplementary file 1 [file Data_Sheet_1.zip › Supplementary Material/The raw data for figure4/ú¿Aú⌐The differential expression of P53 among different small sampling blocks in the same multi-site sampling block/FIGURE 4. (A)-left 1(20x).jpg]

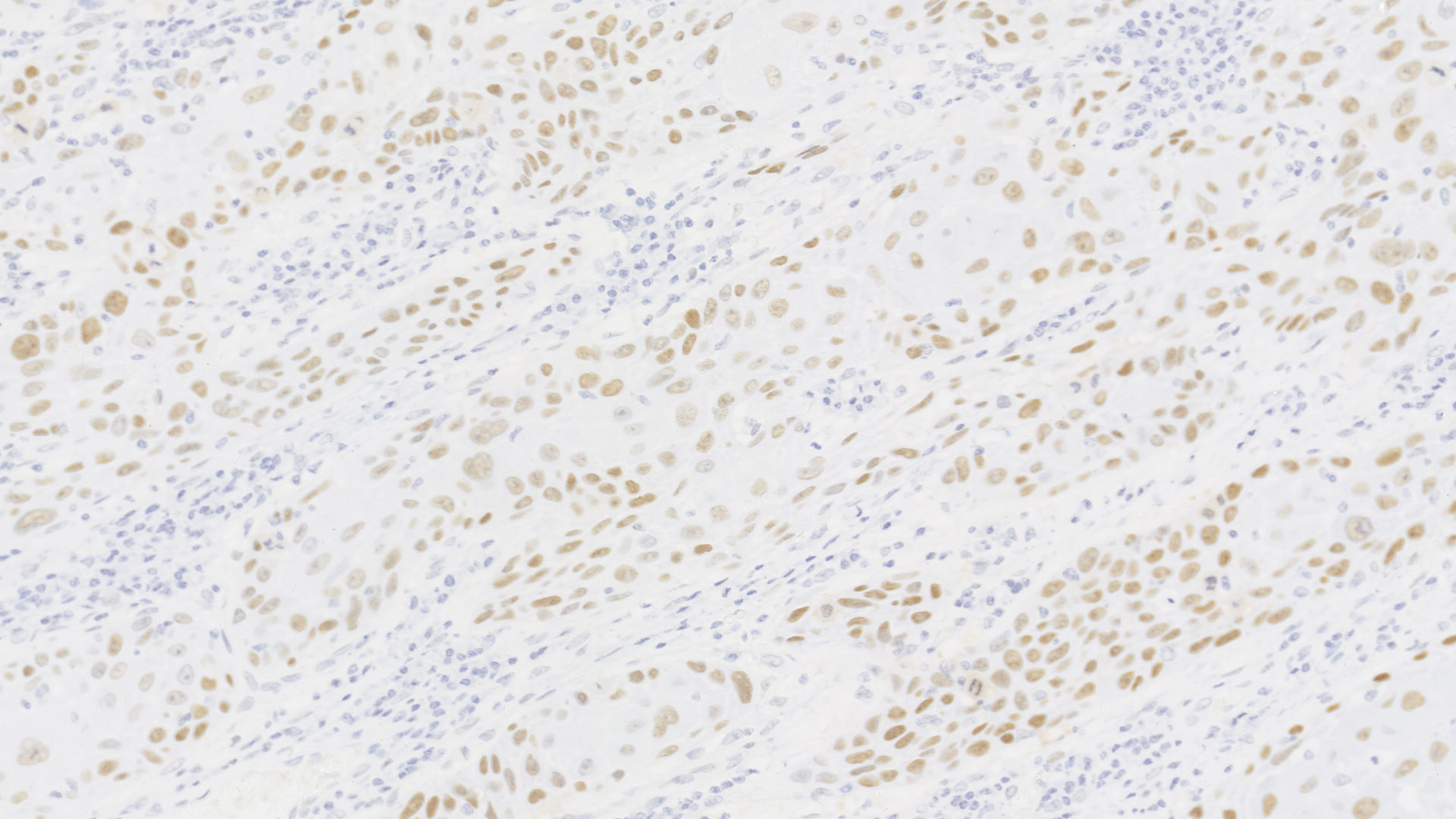

Supplement: Supplementary file 1 [file Data_Sheet_1.zip › Supplementary Material/The raw data for figure4/ú¿Aú⌐The differential expression of P53 among different small sampling blocks in the same multi-site sampling block/FIGURE 4. (A)-left 2(20x).jpg]

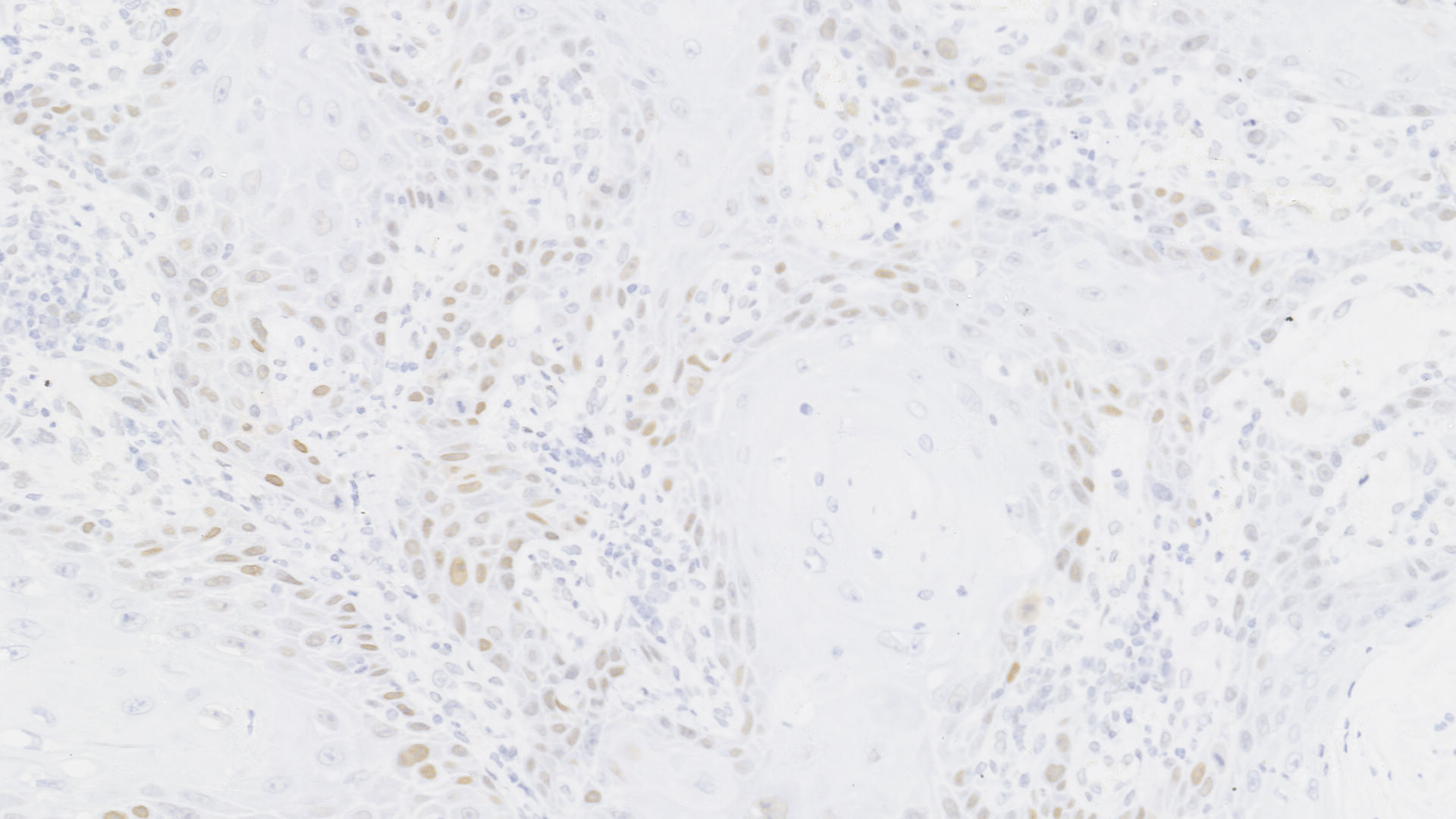

Supplement: Supplementary file 1 [file Data_Sheet_1.zip › Supplementary Material/The raw data for figure4/ú¿Aú⌐The differential expression of P53 among different small sampling blocks in the same multi-site sampling block/FIGURE 4. (A)-left 3 (20x).jpg]

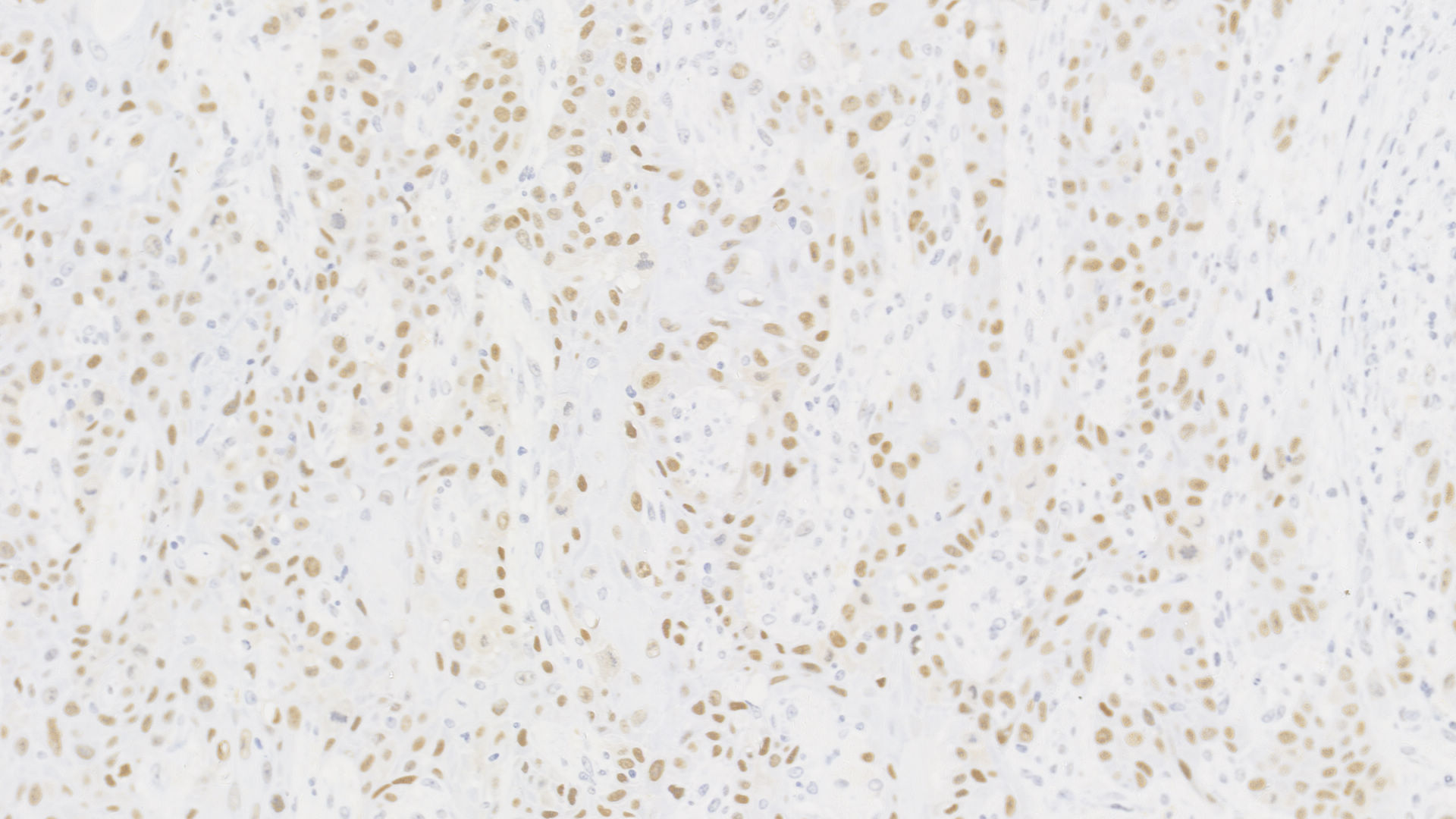

Supplement: Supplementary file 1 [file Data_Sheet_1.zip › Supplementary Material/The raw data for figure4/ú¿Aú⌐The differential expression of P53 among different small sampling blocks in the same multi-site sampling block/FIGURE 4. (A)-right 1(20x).jpg]

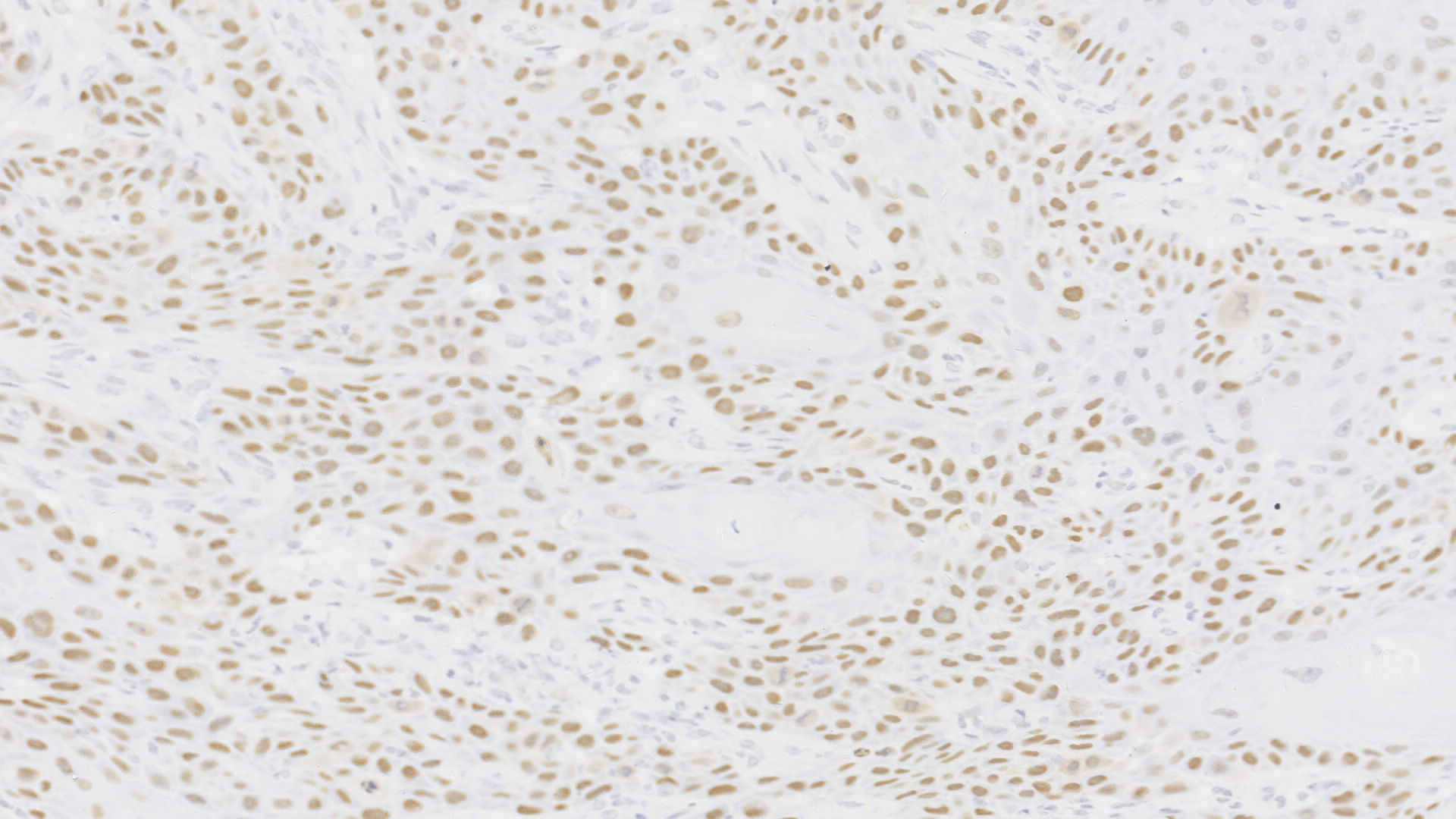

Supplement: Supplementary file 1 [file Data_Sheet_1.zip › Supplementary Material/The raw data for figure4/ú¿Aú⌐The differential expression of P53 among different small sampling blocks in the same multi-site sampling block/FIGURE 4. (A)-right 2(20x).jpg]

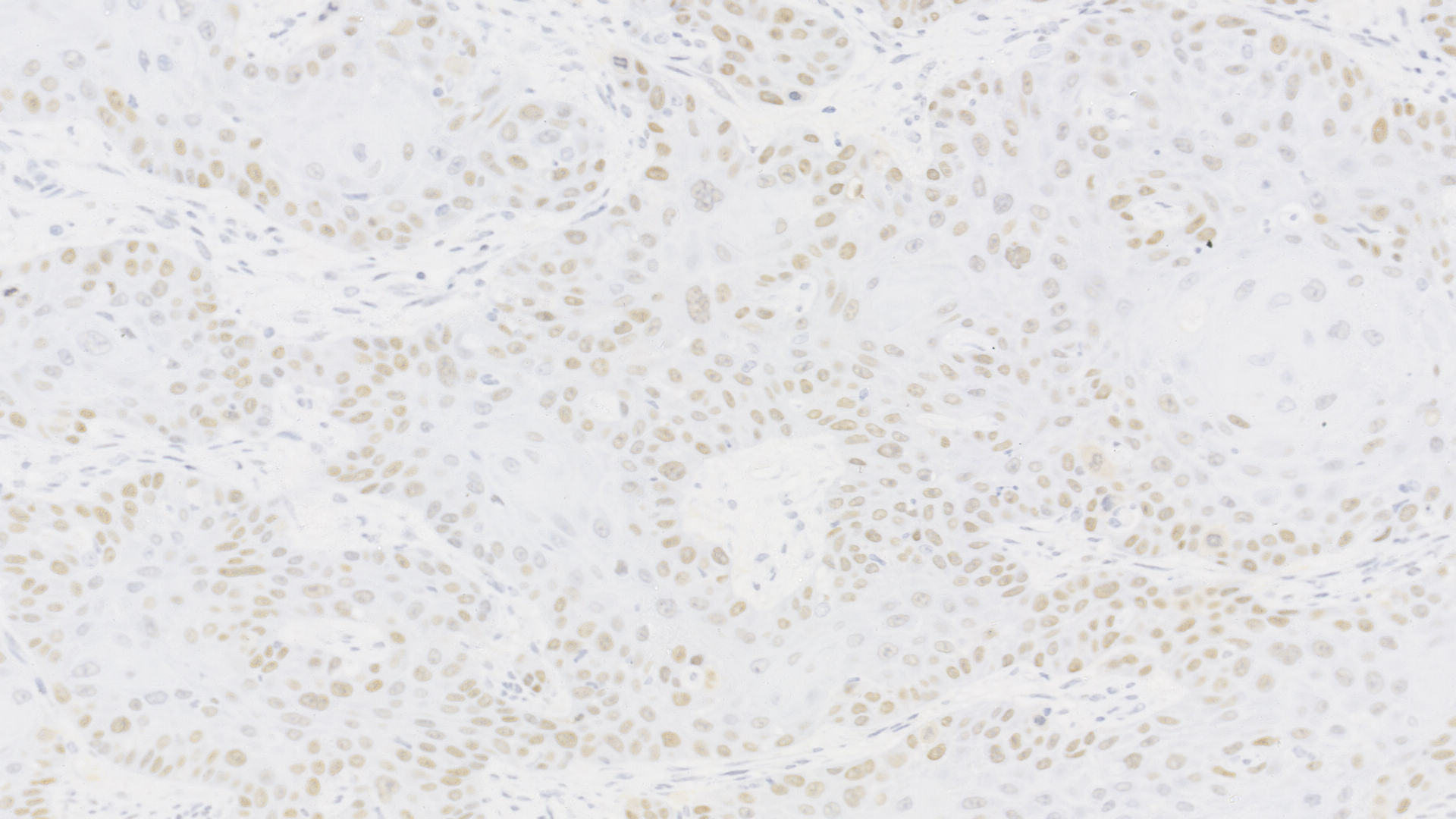

Supplement: Supplementary file 1 [file Data_Sheet_1.zip › Supplementary Material/The raw data for figure4/ú¿Aú⌐The differential expression of P53 among different small sampling blocks in the same multi-site sampling block/FIGURE 4. (A)-right 3(20x).jpg]

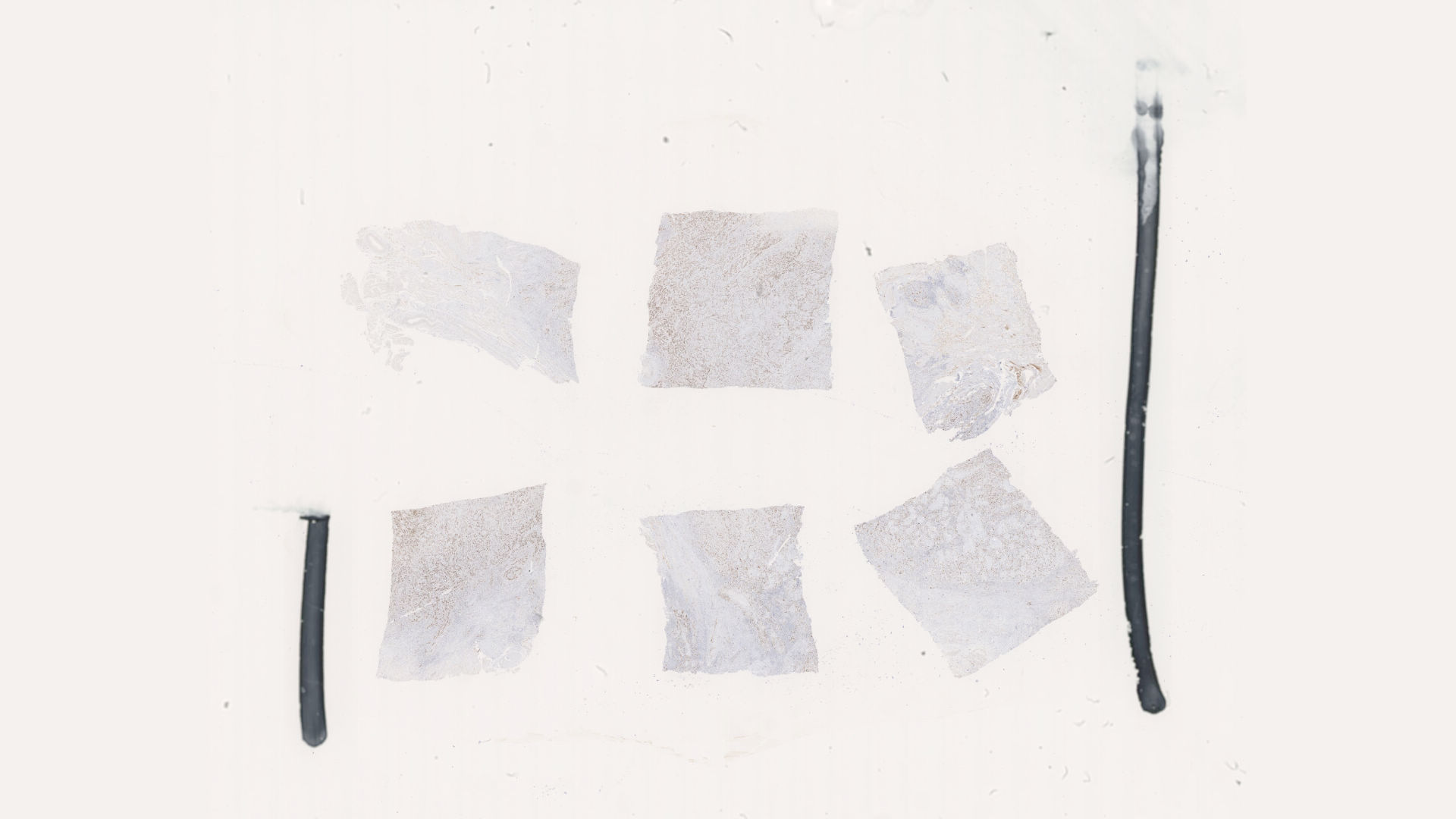

Supplement: Supplementary file 1 [file Data_Sheet_1.zip › Supplementary Material/The raw data for figure4/ú¿Bú⌐The differential expression of Ki67 among different small sampling blocks in the same multi-site sampling block/FIGURE 4. (B)-center(0.48x).jpg]

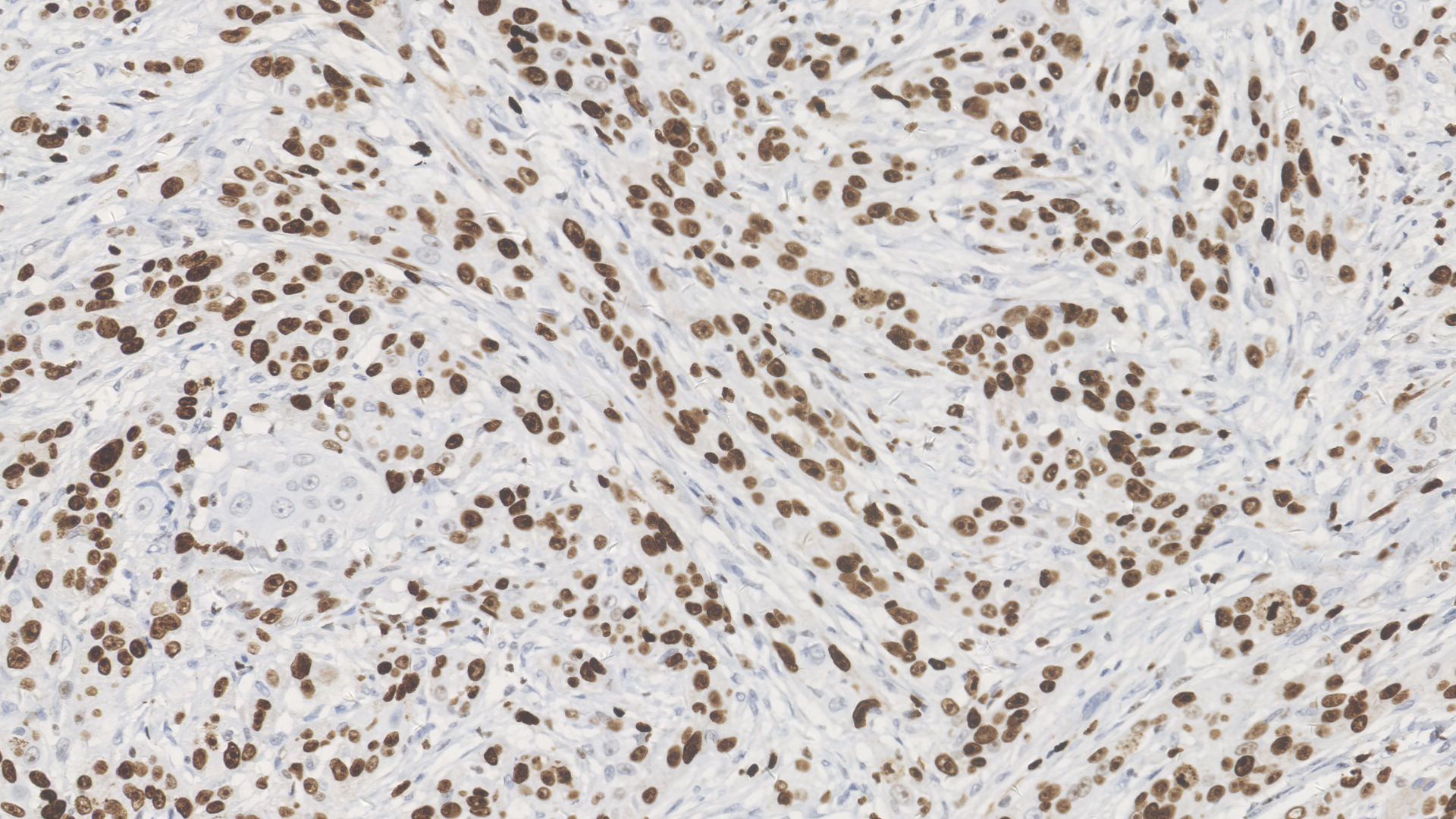

Supplement: Supplementary file 1 [file Data_Sheet_1.zip › Supplementary Material/The raw data for figure4/ú¿Bú⌐The differential expression of Ki67 among different small sampling blocks in the same multi-site sampling block/FIGURE 4. (B)-left 1(20x).jpg]

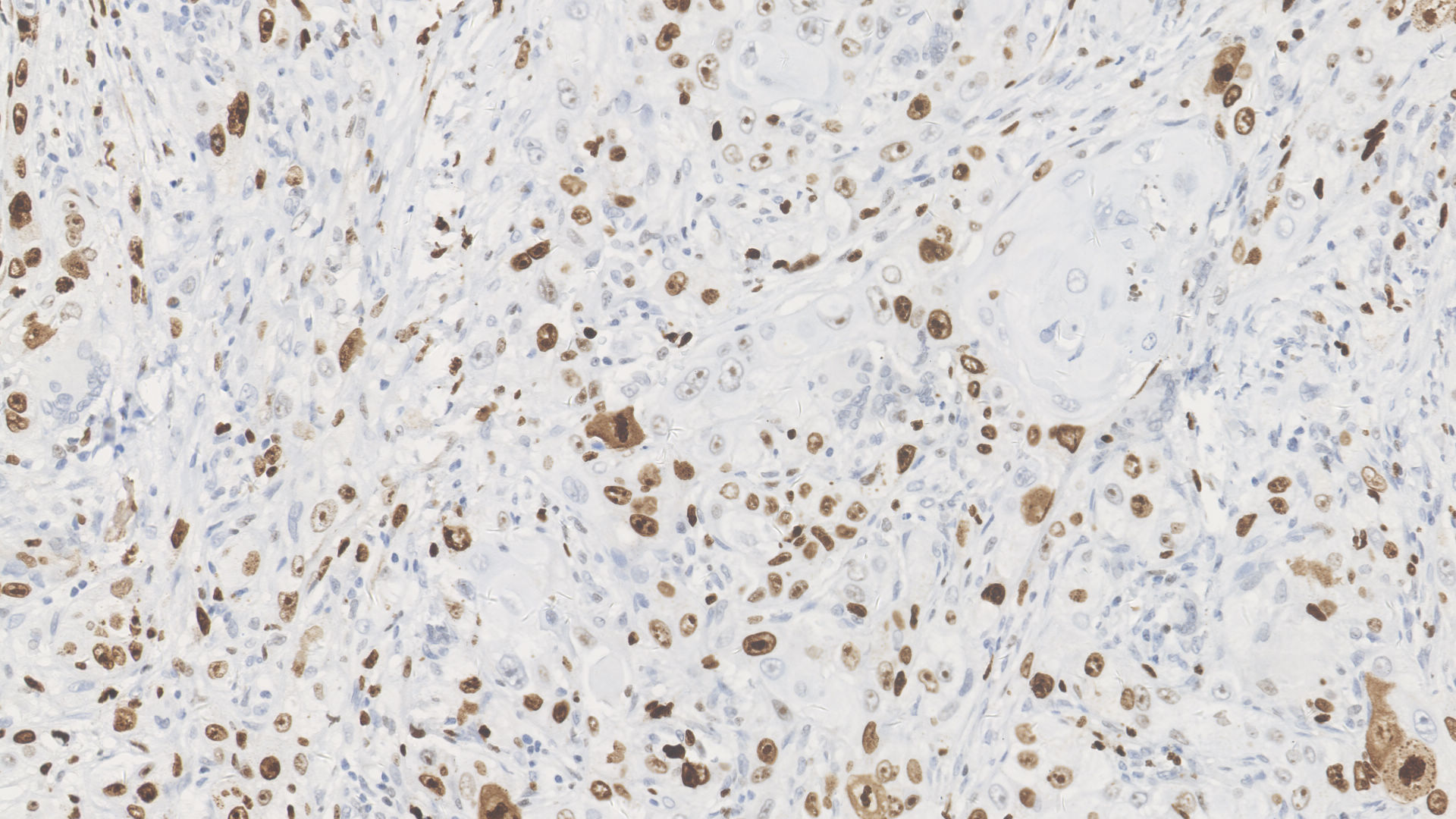

Supplement: Supplementary file 1 [file Data_Sheet_1.zip › Supplementary Material/The raw data for figure4/ú¿Bú⌐The differential expression of Ki67 among different small sampling blocks in the same multi-site sampling block/FIGURE 4. (B)-left 2(20x).jpg]

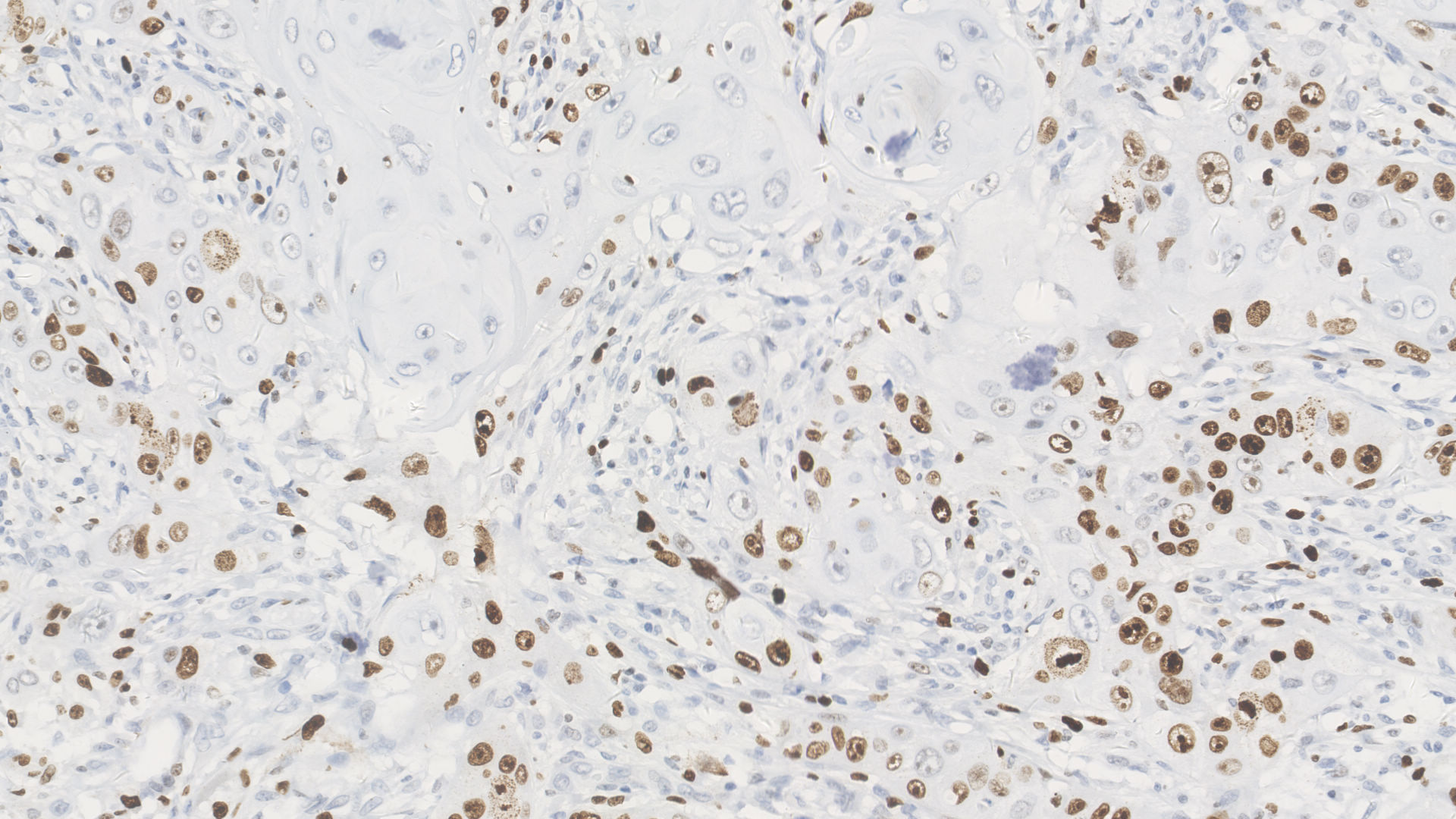

Supplement: Supplementary file 1 [file Data_Sheet_1.zip › Supplementary Material/The raw data for figure4/ú¿Bú⌐The differential expression of Ki67 among different small sampling blocks in the same multi-site sampling block/FIGURE 4. (B)-left 3 (20x).jpg]

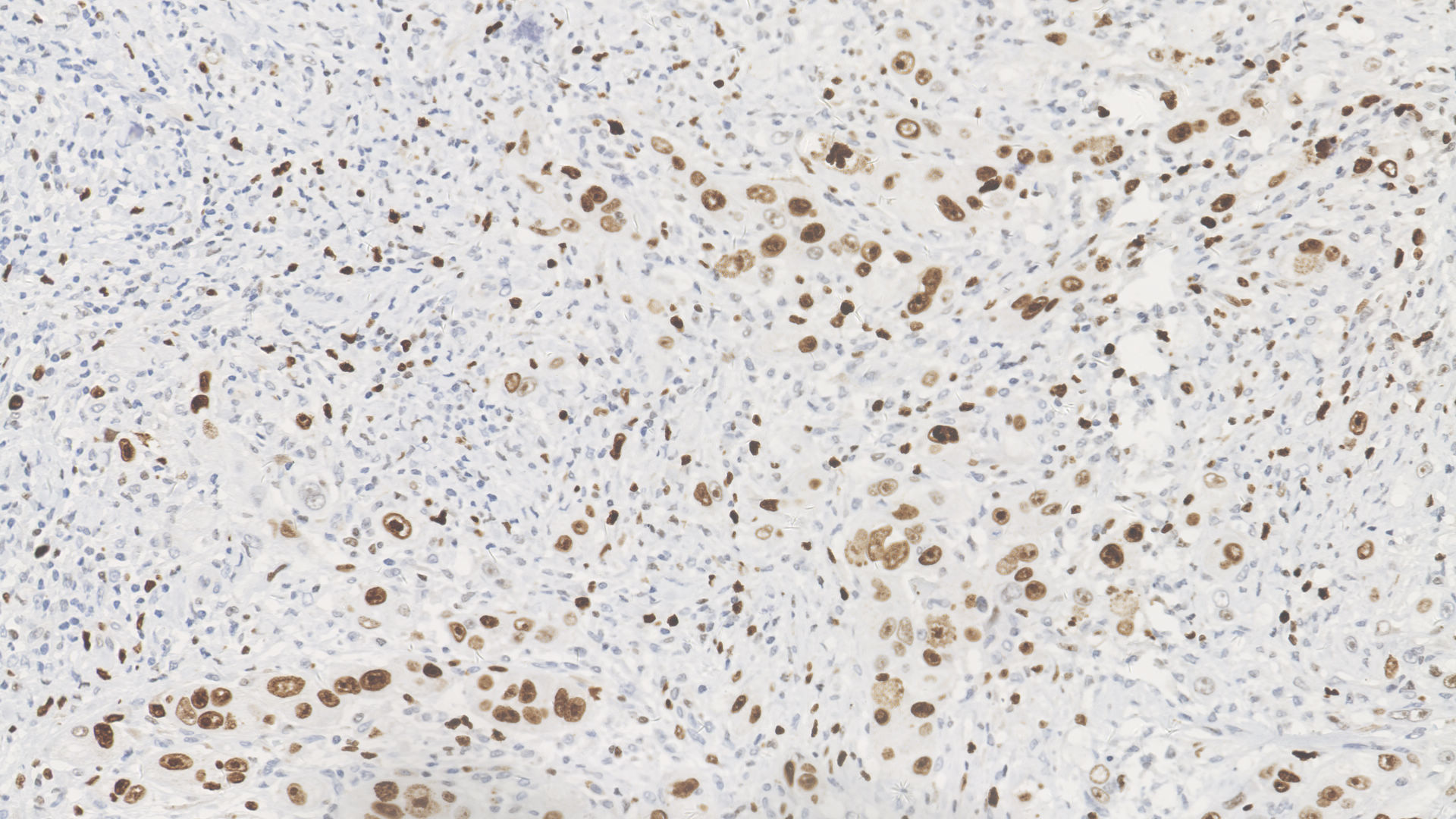

Supplement: Supplementary file 1 [file Data_Sheet_1.zip › Supplementary Material/The raw data for figure4/ú¿Bú⌐The differential expression of Ki67 among different small sampling blocks in the same multi-site sampling block/FIGURE 4. (B)-right 1(20x).jpg]

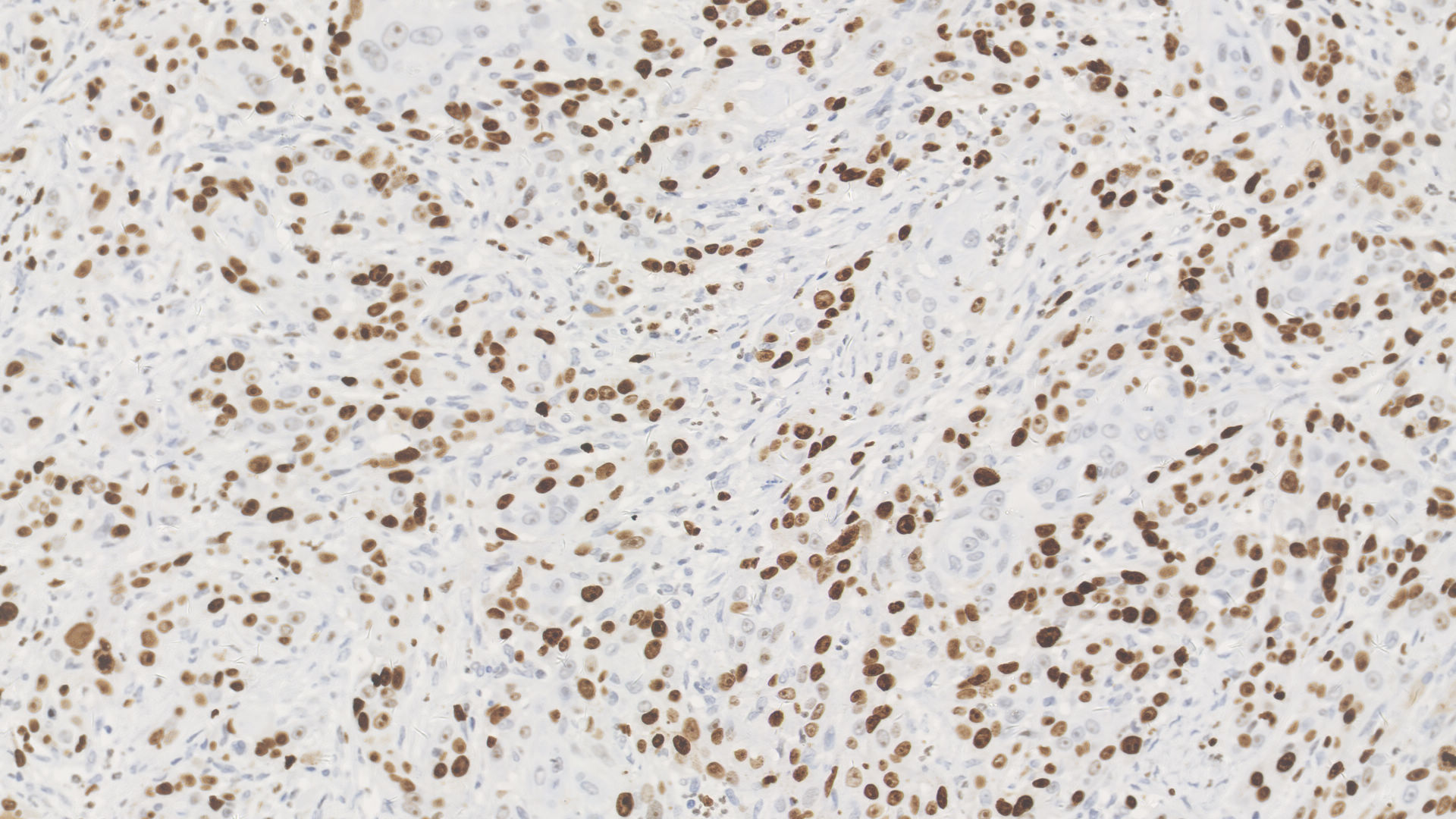

Supplement: Supplementary file 1 [file Data_Sheet_1.zip › Supplementary Material/The raw data for figure4/ú¿Bú⌐The differential expression of Ki67 among different small sampling blocks in the same multi-site sampling block/FIGURE 4. (B)-right 2(20x).jpg]

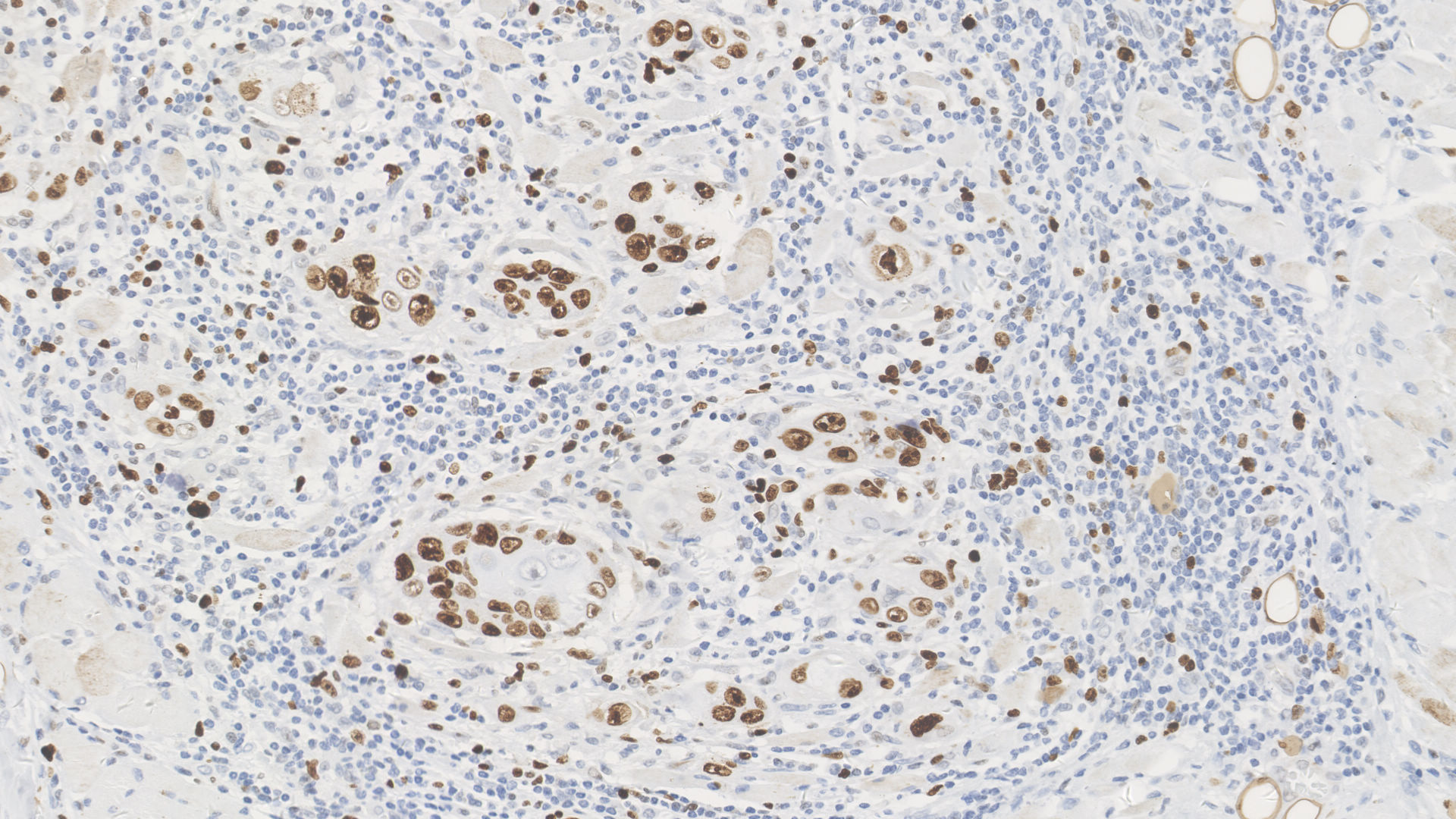

Supplement: Supplementary file 1 [file Data_Sheet_1.zip › Supplementary Material/The raw data for figure4/ú¿Bú⌐The differential expression of Ki67 among different small sampling blocks in the same multi-site sampling block/FIGURE 4. (B)-right 3ú¿20xú⌐.jpg]
